# Robust estimation of diagnostic rate and real incidence of COVID-19 for European policymakers

**DOI:** 10.1371/journal.pone.0243701

**Published:** 2021-01-07

**Authors:** Martí Català, David Pino, Miquel Marchena, Pablo Palacios, Tomás Urdiales, Pere-Joan Cardona, Sergio Alonso, David López-Codina, Clara Prats, Enrique Alvarez-Lacalle

**Affiliations:** 1 Department of Physics, Universitat Politècnica de Catalunya (UPC ⋅ BarcelonaTech), Barcelona, Spain; 2 Comparative Medicine and Bioimage Centre of Catalonia (CMCiB), Fundació Institut d’Investigació en Ciències de la Salut Germans Trias i Pujol (IGTP), Badalona, Catalonia, Spain; 3 Experimental Tuberculosis Unit (UTE), Fundació Institut d’Investigació en Ciències de la Salut Germans Trias i Pujol (IGTP), Universitat Autònoma de Barcelona (UAB), Badalona, Catalonia, Spain; 4 Centro de Investigación Biomédica en Red de Enfermedades Respiratorias (CIBERES), Madrid, Spain; Agencia de Salut Publica de Barcelona, SPAIN

## Abstract

Policymakers need clear, fast assessment of the real spread of the COVID-19 epidemic in each of their respective countries. Standard measures of the situation provided by the governments include reported positive cases and total deaths. While total deaths indicate immediately that countries like Italy and Spain had the worst situation as of mid-April, 2020, reported cases alone do not provide a complete picture of the situation. Different countries diagnose differently and present very distinctive reported case fatality ratios. Similar levels of reported incidence and mortality might hide a very different underlying pictures. Here we present a straightforward and robust estimation of the diagnostic rate in each European country. From that estimation we obtain a uniform, unbiased incidence of the epidemic. The method to obtain the diagnostic rate is transparent and empirical. The key assumption of the method is that the infection fatality ratio of COVID-19 in Europe is not strongly country-dependent. We show that this number is not expected to be biased due to demography nor to the way total deaths are reported. The estimation protocol is dynamic, and it has been yielding converging numbers for diagnostic rates in all European countries as from mid-April, 2020. Using this diagnostic rate, policy makers can obtain Effective Potential Growth updated every day, providing an unbiased assessment of the countries at greater risk of experiencing an uncontrolled situation. The method developed has been and will be used to track possible improvements in the diagnostic rate in European countries as the epidemic evolves.

## Introduction

The evolution of the epidemic in Europe has affected Spain and Italy more strongly than other countries so far. This is clear from reported cases and fatalities in these countries [1–3]. However, comparative assessment of the spread of the pandemic in other European countries has been more difficult to make. The reason is that the real incidence of the epidemic in each country cannot be known with certainty, because countries are not able to perform the same number of polymerase chain reaction tests (PCR) and consequently the comparison of the ratio of those infected is difficult [4]. Policy responses have also differed, with some countries focusing on clinical testing in hospitals, while others have tried to use tests, at least partially, to determine some local chains of transmissions [5, 6]. The lack of clear inter-country comparison in Europe has deep implications for the future structure of the European Union since many decisions are taken based upon the sense of gravity in the country in question. For these reasons, it is important to have a proper measure of the relative spread of the epidemic. Policymakers must know what the real situation in their own countries is in comparison to others so that their decisions on the future of reopening and economic reconstruction are taken based not on false impressions, but on data. In this sense, policymakers must perceive the method as unbiased, simple, and robust. Most importantly, the relative comparisons between countries must be as shielded as possible from the hypothesis of the method. In this sense, methods have recently been developed [7–9] in order to assess the situation inferred from data. This work has been instrumental in providing a better picture of the situation. However, the work lacks the recipe-type nature needed to direct a policy response.

The focus of this paper is, therefore, to introduce a method to compute the real diagnostic rate and the real incidence of COVID-19 in each European country, testing whether the key hypothesis of the method is fulfilled and, if slightly off, whether it would affect all countries in the same way. In other words, we provide a recipe for policymakers that we have shown to be correct, and unbiased across countries and useful to make inter-country comparison, provided the evolution and prognosis of the disease in a patient is not strongly dependent on socio-economic factors, and only on age, sex and previous clinical history.

We must recall here that the ability to determine the diagnostic ratio is essential to evaluate what the real number of infected people is. Knowledge of this number is not only useful to visualize the full scope of the epidemic but also to properly estimate the number of people with probable short-term immunity. In this sense, our method can be added as an empirical take on other assessments of the real incidence of the disease and to study the possibility of developing herd immunity. A large number of real infected people would be a positive scenario for policymakers while a low number would be negative. It is thus very important to err on the side of caution in all our estimates, settling on the less optimistic take.

The basic structure of the paper is the following. First, we give a general overview of our framework in the methods section. Then we discuss our key assumption: the infection fatality ratio (IFR) in European countries experiencing a significant incidence will be roughly the same, given the similar structure of the population. If IFR were to be lower, or higher, it would affect all countries in the same way and would not affect most policy decision-making since it would move all countries in the same direction. We take this IFR to be 1% and proceed to test whether, effectively, there is a strong correlation between the day of reported deaths with the number of cases taken 7-10 days before. Once a given value for the IFR is taken, one must consider that people do not die immediately from the disease, as it takes roughly 18 days after infection [10–12]. In other words, the present values of the death toll can provide an estimation of the number of infected people 18 earlier. Knowing the number of infected people at present, and not 18 days in the past, is crucial. We attack this problem considering that people who become infected are usually diagnosed a few days after the onset of the symptoms, which can be 8 to 14 days after infection occurs. By comparing the number of people diagnosed on a certain date with our estimation of the real number of infected people, we can estimate what percentage of the cases is being diagnosed. We can then calculate this for different countries and regions and test how this ratio has changed dynamically as the epidemic advanced.

In the results section, we provide a full detailed description of how this fraction has become steady in the last weeks. We demonstrate that the percentage of diagnosis throughout the development of the epidemic has taken values that gradually converge for most countries. This gives a final clear picture showing the rate of diagnosis for each country. Using this rate it is straightforward to provide a present-day estimate of the incidence given the number of reported infected people in each country as long as we can observe that the rate of diagnosis remains fairly constant. For policymakers, we have constructed an index called Effective Potential Growth (EPG) that combines this information with the growth rate of the epidemic to provide insight regarding which countries are, comparatively and in the short-term, in the potentially most complicated situation [9]. Finally, we analyze the sensitivity of the EPG index to variations of the different parameters not based in previous studies and we show how robust is the index in predicting and increase of the incidence during the first phase of the pandemic and in detecting secondary outbreaks.

## Methods

### Framework of our methodology

**Fig 1:**
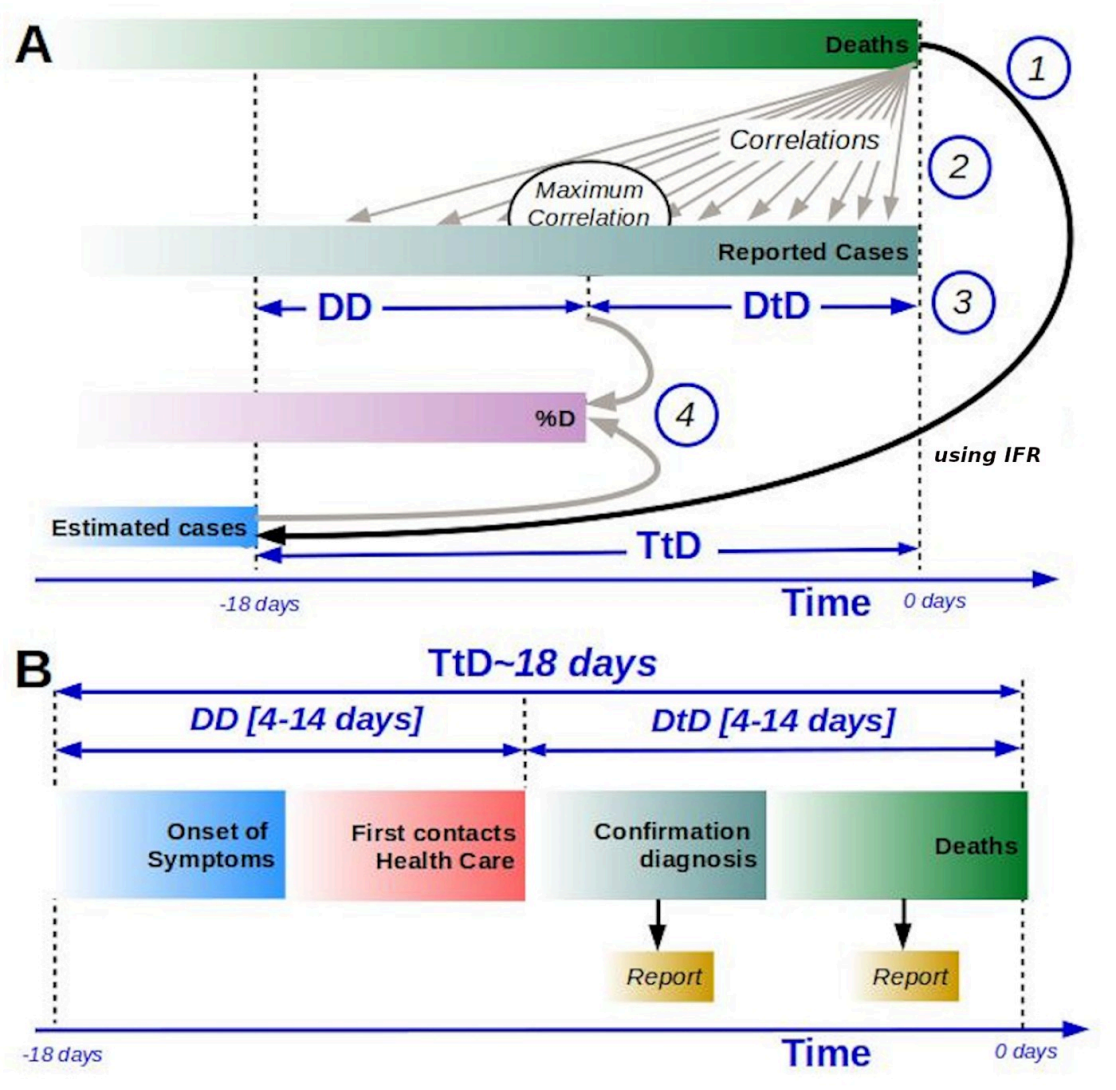
General framework of the calculation of the percentage of diagnosis. (A) Processes involved in the calculation of the percentage of diagnosis: 1. Evaluation of the estimated cases using TtD and IFR, 2. Calculation of time correlation between reported cases, *C*_*t*_, and reported deaths, *d*_*t*_, 3. Evaluation of the time between diagnosis and death (Diagnosis to Death DtD) by the maximum of correlation (country dependent), with DD as the Delay to Detection, and 4. Evaluation of percentage of diagnosis based on estimated cases and reported cases. (B) Standard evolution of casualties by COVID-19, from the onset of the symptoms to death; times to reported cases and deaths are shown. Time-lines in the figure are not proportional to real-time delays.

Our analysis will be applied to European countries with a minimum of 500 deaths on April 15, 2020 so that we can guarantee a minimum statistical significance, so that the fluctuations $1/\sqrt{N}$ of an associated binomial distribution are below 5%. The analyzed countries are: Belgium, France, Germany, Italy, Netherlands, Portugal, Spain, Sweden, Switzerland and the United Kingdom. Our two core assumptions are that the IFR in all European countries is roughly the same and that reported data of death due to COVID-19 is uniform in all European countries under consideration. We will address these two hypotheses in the following sections. With these assumptions we need to carry out four steps, as indicated in Fig 1, to obtain the percentage of diagnosis. First, using a common reference IFR = 1% and, given the reported death count, we estimate the number of cases 18 days ago. According to medical reports people die between 15 and 22 days after the development of the first symptoms [13]. This time to death, TtD = 18 days, after the development of the first symptoms will not be country-specific for demographic reasons. The estimated number of infected people with the disease at time *t*, *E*_*t*_, (see process in Fig 1(A)) reads:

$$\begin{array}{c} E_{t}=\frac{d_{t+\text{TtD}}}{\text{IFR}}, \end{array}$$ (1)

where *d*_*t*+TtD_ is the number of reported deaths at time *t* + TtD.

This allows us to estimate the number of cases TtD = 18 days ago. This value can be compared with the number of cases detected 18 days ago, obtaining a diagnostic percentage. This result is an unrealistic lower bound because no one performs PCR testing the first day of symptoms; this is usually done much later. Actually, people normally do not call a doctor at the first symptoms. Furthermore, depending on the availability of tests, saturation of the health system, and other external factors, countries show great variability in the time of diagnosis delay.

Countries accumulate some delay that may reach 18 days, in the case of a country detecting people as late as death. This delay to detection (DD) due to lags in diagnosis corresponds to the time between the patient having the first symptoms and being reported by the health system. In fact, this time in some countries may vary throughout the course of the infection. Therefore we cannot assume that the estimated and the reported data are comparable and we need to know what the diagnostic time was for each of the countries studied.

We can compare the reported deaths with the reported cases to find the maximal correlation; see process 2 in Fig 1(A); to estimate the DD, see process 3 in Fig 1(A). Finally the ratio between reported cases at DD and estimated cases, see below, provides an estimation of the percentage of diagnosis; see process 4 in Fig 1(A). Note that the usual development of the reporting of a new case/death, see Fig 1(B), depends on the particular country under consideration, which determines DD. In fact, DD also includes a delay in reporting, from the diagnosis to death, to official information systems.

### IFR of COVID-19 in Europe

The cornerstone of our analysis is that the IFR in all European countries will not be biased against any country in particular. We should point out immediately that we are not arguing that there are not important uncertainties in the IFR. What we do claim and check in this methodology is that these uncertainties will not generate any biased against particular countries and should not affect policy decision. We take the IFR of COVID-19 in Europe to be between 0.3-3% and we assume 1% to be the benchmark scenario.

This value (1%) is the case fatality ratio (CFR) observed in the initial stages of the pandemic in China, Republic of Korea (South Korea) and the Diamond Princess cruise. In these three locations, it was found to be around 1-2.6%, and error margins came from different sources, respectively [14–16]. In China, once adjusted for demography and under-ascertainment, case fatality ratio during February, 2020 was estimated to be 1.4%. In South Korea, the ability to test all the population in contact with infected people and the tracking of contagious chains was thorough. Despite this, the reported CFR increased from initial values around 0.5-0.7% to higher values around 2%. In the Diamond Princess cruise, CFR for confirmed cases was 2% but estimation of false negatives and the possibility that a fraction of the passengers never developed symptoms and was never tested put the CFR again around 1%. South Korea and the Diamond Princess cruise provide complementary evidence, one coming from a natural experiment and another from a country with the ability to perform half a million tests/day from the very beginning of the transmission chain [17]. If we accept the two measurements of the CFR independently, the most likely interval of IFR is between 0.5 and 2%.

Experimental results from random testing in the German city of Gangelt [18] and preliminary results from Iceland [19, 20] indicate the presence of a layer of people fully asymptomatic people that are normally not detected. This group of people have passed the disease without any knowledge seems to be larger than previously thought. These preliminary studies point to a CFR of around 0.5% in zones where the epidemic was not fully spread. We cannot disregard the possibility that, just as CFR increased with time even in South Korea, similar studies in countries with more cases, could reveal a higher CFR.

Recently, other authors, using different techniques, have obtained estimates for CFR or IFR in different countries/regions. [21] obtained the cumulative incidence of SARS-CoV-2 infection in New York State based on dry-blood spot SARS-CoV-2 antibody reactivity performed on 15,000 individuals over 18 years from March 19, 2020 to 28 March, 2020. They found that the estimated cumulative incidence was 14% (2.7 million people), which gives a CFR of 0.5%. [22] uses a mathematical model with a cohort analysis approach to determine the range of case fatality ratios in Hubei province (China) from January 22, 2020 to March 11, 2020. They demonstrated that CFR is from 4.8% to 6.1%. [23] statistically estimate the incidence of the pandemic in 70 countries by using indirect reporting, where the questions a participant answers are not about herself (*network scale-up method*). They found Brazil, Ecuador and Ukraine CFR are 1% on May 17, 1.61% on April 15 and 0.56% on April 26, 2020, respectively. Finally, [24] analyze data from 139 countries to conclude that the global IFR is 1.04%.

It is thus reasonable to consider IFR at 1% as an easy policy guiding principle and not to use the more positive scenario of 0.5%.

### Unbiased nature of IFR in Europe

There are three sources of possible biased IFR across countries. The disease affects older people with comorbidity problems more than to healthy younger ones, and men diagnosed with COVID-19 are more likely to die than women [25], the same way it happened in previous coronavirus epidemics [26, 27]. Across all European countries, the male/female ratio is approximately the same except for the oldest people. For people above 80 years, sex ratio (number of men by 100 women) in Europe oscillates between 85 (Albania) and 33 (Latvia) [28]. This is precisely the group with the highest case fatality ratio. It is thus very important to assess how the different demographic structures of European countries could affect our central benchmark [29]. The same must be said about the relative prevalence of other comorbidity factors. We proceed to show that, with the data we have today, and the demographic and comorbidity structures, none of these possible sources of bias can have anything but a small effect. To do so, we will use and make a comparison with the CFR of South Korea on April 15, 2020, of 2.16%.

Table 1 shows, for each analyzed age group, the demographic structure of South Korea, the number of cases and deaths, the corresponding percentage, and the corresponding CFR officially reported on April 15, 2020. As may be observed in the table, by comparing the percentage of population and cases for all the age groups we can conclude than in South Korea people below 49 years old are infected/detected less than their corresponding population importance. The contrary occurs for the people above 50 years old. People over 80 present an increase in infection of 32% with respect to their population importance. This is probably because they present increased symptoms and are therefore tested more often. However, recent studies [30, 31] show that younger people are less susceptible to be infected. Regarding deaths, these differences are even more important, and the change occurs for the age group 60-69.

**Table 1:** For each age group: Number of cases and deaths reported in South Korea on April 15, 2020; percentage of population, COVID-19 cases and deaths, and case fatality ratio.

*Age group*	0-9	10-19	20-29	30-39	40-49	50-59	60-69	70-79	≥80
*Cases*	136	576	2909	1135	1411	1942	1342	704	480
*Deaths*	0	0	0	1	3	14	33	68	111
*Population (%)*	8.1	9.3	13.1	13.8	16.0	16.5	12.6	6.9	3.6
*Cases (%)*	1.3	5.4	27.4	10.7	13.3	18.3	12.6	6.6	4.5
*Deaths (%)*	0	0	0	0.4	1.3	6.1	14.4	29.6	48.3
*CFR (%)*	0	0	0	0.09	0.21	0.72	2.46	9.66	23.13

Source: United Nations World Population Prospects 2019 and Korea Centers for Disease Control and Prevention.

To analyze what the role played by the differences in demography in Europe in the COVID-19 cases and fatalities is, we have obtained from the United Nations World Population Prospects 2019 the demographic distribution by age in the considered countries (see Table 2).

**Table 2:** Percentage of population by age group for the analyzed European countries.

*Country/Age group*	0-9	10-19	20-29	30-39	40-49	50-59	60-69	70-79	≥80
*Belgium*	11.2	11.3	12.0	13.0	13.1	13.8	11.8	8.1	5.7
*France*	11.5	12.1	11.3	12.3	12.8	13.2	11.9	8.8	6.2
*Germany*	9.4	9.5	11.2	13.0	12.2	16.1	12.7	8.9	7.0
*Italy*	8.3	9.5	10.1	11.6	14.9	15.8	12.4	10.0	7.5
*Netherlands*	10.2	11.4	12.2	12.2	12.6	14.7	12.4	9.3	4.9
*Portugal*	8.2	10.0	10.5	11.9	15.5	14.5	12.7	10.0	6.7
*Spain*	9.1	10.1	9.9	12.6	17.0	15.1	11.4	8.6	6.3
*Sweden*	11.8	11.2	12.6	13.1	12.5	12.8	10.8	9.8	5.3
*Switzerland*	10.2	9.6	12.0	14.1	13.5	15.3	11.3	8.7	5.3
*United Kingdom*	11.8	11.3	12.6	13.7	12.7	13.5	10.7	8.6	5.1

Source: United Nations World Population Prospects 2019.

We can readily assess that, compared with South Korea, all the countries have a larger percentage of population over 70 years. The percentage is even larger if people over 80 is considered (108% larger for Italy). It is also important to note that the relative differences in each of the cohorts between the European countries shown in the table is small. Only Italy presents a relevant larger than average ratio of people over 80.

To analyze the role played by demography, we use this demographic data and the number of cases and deaths reported by South Korea on April 15, 2020 (see Table 1) to estimate the CFR corrected by population for each European country. To obtain it, first, we estimate the number of cases for each European country *E* and age group *A* as follows:

$$\begin{array}{c} \text{Estimated}\;\text{Cases}\;\text{in}\;E_{A}=\text{Cases}\;\text{South}\;{\text{Korea}}_{A}\times \frac{\%\text{Population}\;E_{A}}{\%\text{Population}\;\text{South}\;{\text{Korea}}_{A}}, \end{array}$$

and the same for the corresponding number of deaths. That is, we are assuming that the CFR for each age group of all the European countries is the same as shown in Table 1 for South Korea. However, this doesn’t imply necessarily that the country CFR for a European country is the one reported by South Korea because the number of cases and deaths for each age group and country is different in each country. Table 3 shows the CFR obtained by performing this analysis and the CFR officially reported at the different European countries on the same date. Both values are presented relative to the CFR reported by South Korea on April 15, 2020, of 2.16%.

**Table 3:** Estimated relative CFR assuming these countries have the same CFR by age group as reported by South Korea on April 15, 2020 (see Table 1), and officially reported relative CFR on that date. CFR of South Korea on April 15, 2020 was 2.16%. The officially reported CFR on that date for each country is indicated in parentheses. Source: European Center for Disease Prevention and Control.

*Country/Relative CFR*	Estimated from South Korea reported data on April 15, 2020	Reported on April 15, 2020
*Belgium*	1.35	6.18 (13.4)
*France*	1.47	7.02 (15.2)
*Germany*	1.56	1.18 (2.6)
*Italy*	1.67	6.00 (13.0)
*Netherlands*	1.29	4.97 (10.7)
*Portugal*	1.56	1.50 (3.3)
*Spain*	1.46	4.63 (10.0)
*Sweden*	1.35	4.17 (9.0)
*Switzerland*	1.31	1.62 (3.5)
*United Kingdom*	1.28	6.93 (15.0)

As may be observed in the first column, when we only consider demographic differences between countries, the difference between the worst and best case of the relative CFR is around 30%. Most countries are in the range between 1.28% and 1.5%, with the average of the relative CFR being 1.34%. Therefore, CFR for all the countries except Italy is, at most 20% from the average value and typically around 10%.

### Analysis of possible bias in reported deaths due to COVID-19

An unrelated source of bias in the estimation of the real COVID-19 cases, is the possibility that different countries differ in how they treat and count the population that dies after having a very bad prognosis. We know this group is strongly affected by the virus [32]. In blunt terms, we must examine the possibility that different countries are counting the raw number of deaths differently.

Before entering in the details of the analysis, let us point out that two indications go against this possibility. First, health care systems in Europe have different resources in different countries with differing focuses and priorities, but they attend anyone with COVID-19 with the exception of possible patients with multifactorial problems who might be in very fragile conditions. Elderly people in nursing homes who die under suspicious situations are uniformly not reported following European Center for Disease Prevention and Control (ECDC) advice. There is a single exception that we know of: Belgium [33]. Belgium seems to be reporting unconfirmed cases from nursing homes without tests as due to COVID-19. It is quite clear that this includes a good number of people who, either, did not die from COVID-19 or for whom COVID-19 was not an important factor in the prognosis. Therefore, we will include a reminder that Belgian data are biased compared with other countries, being 48% lower on April, 20 2020 [34], given the number of reported deaths from nursing homes compared with hospitals. There is a second argument regarding the treatment of the elderly population in other countries. If large undercounting is the case, it should be noted in the case fatality ratio for people 80 years and older, which is not observed in the countries from which we have data.

Regarding the possible COVID-19 mortality not reported in the analysis due to a lack of PCR tests, we analyze here the situation in Spain. The National Epidemiology Center (Instituto de Salud Carlos III) of Spain published the results of the Daily Mortality Monitoring System (MoMo) for June 17, 2020 [35]. They evaluated which periods had mortality well above the average for previous years. When evaluating the period from March 13 to May 22, 2020 for the whole of Spain, they saw that, as expected, mortality was much higher than in previous years. An increase of 55.8% was observed. However, it is interesting to compare this with the data reported for COVID-19 deaths. The reported deaths by COVID-19 were roughly 28,000 and the reported excess of deaths by the MoMo surveillance system was 44,000. We think that the assessment of around 35% underreporting can be taken indeed as a worst-case scenario for a highly impacted country. It seems reasonable to expect other countries to have underreported at around this level 15-35% [36, 37]. All the data suggest right now, that the undercounting due to a different treatment of the very fragile population is highly unlikely across Europe, and at most introduces changes in IFR of ±10%.

### Treatment of inter-country bias in diagnostic time-delays

**Fig 2:**
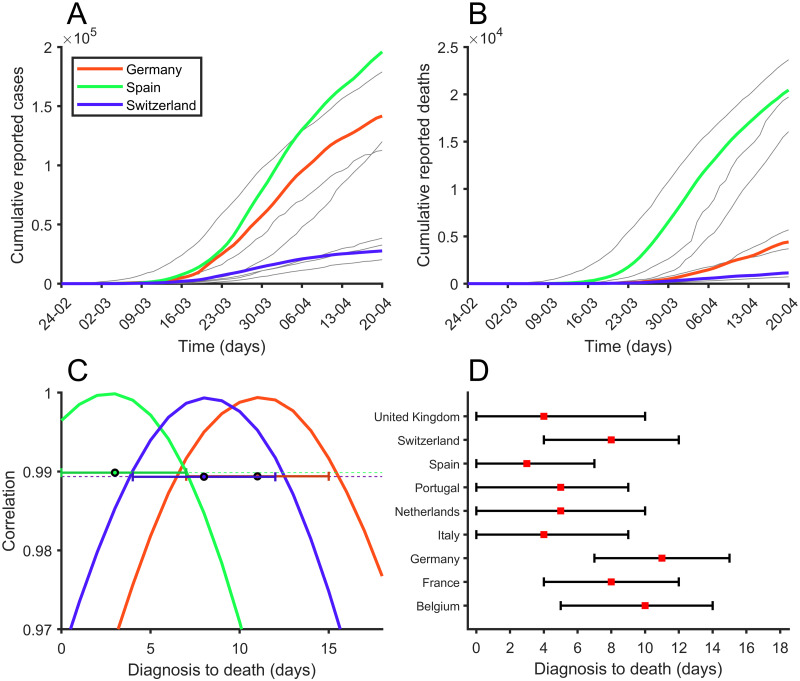
Correlation between reported cases and deaths. (A) Number of cumulative reported cases, (B) Number of cumulative reported deaths and (C) Correlation between reported cumulative cases and reported cumulative deaths exploring different delays between diagnosis (reported) and death, for Germany (red), Spain (green), and Switzerland (blue). (D) Maximum correlation is marked with a red square for each country. 99% correlation interval can be seen with black bars.

Having shown that the IFR should not represent a bias in European countries larger than 25%, we now address the question of how to deal with the real sources of bias in the diagnostic rate for each country. To estimate DD we look for a correlation between the number of reported cases (see Fig 2A) and the number of reported deaths (see Fig 2B) [1, 38]. To deal with noise effects we put a weighted moving average filter on the data both of cases and deaths. The correlation time between reported cases and reported deaths will be called time from diagnosis to death (DtD), and:

$$\begin{array}{c} \text{TtD}=\text{DD}+\text{DtD}. \end{array}$$ (2)

In Fig 2C we see the correlation [39] between reported cases and reported deaths assuming different DtD for Germany, Spain, and Switzerland. As you might expect, correlations have values close to 1. In most cases the correlation has a concave parabolic shape with a clearly defined maximum. We assume this maximum represents DtD for each country. The correlation interval is estimated as the points where the correlation is greater than 99% of the observed maximum. We decided to set a lower limit of 4 days and an upper limit of 14 days [11] because we believe that time outside this range would be unrealistic. For countries that have not seen a clear correlation (Sweden) it was decided to explore the entire DtD interval (4 to 14 days). Approximate values for DtD are shown in Fig 2D. In the S1 File the correlation curves can be seen for the 10 analyzed countries (see S1 Fig in S1 File). Then, by using Eqs (1) and (2), the percentage of diagnosis cases diagnosed at time *t*, %*D*_*t*_, reads:

$$\begin{array}{c} \%D_{t}=\frac{E_{t-\text{DD}}}{C_{t}}=\frac{d_{t+\text{TtD}-\text{DD}}}{\text{IFR}\;\;C_{t}}=\frac{d_{t+\text{DtD}}}{\text{IFR}\;\;C_{t}}, \end{array}$$ (3)

where *C*_*t*_ is the number of reported cases at time *t*.

## Results

### Diagnostic rate by country

As discussed in Methods, we use the same IFR = 1% in all European countries instead of making small corrections for demography. The bias due to demography was shown to be around 10%-15%, precisely the same order of magnitude we obtained for the possible bias in the counting of reported mortal cases. Given that our aim was to provide a clear method for policymakers and that there are no data on how, or even if, the two correlate, a common IFR allows us to homogenize the results with the clear limitation that we will obtain reasonable estimations and not exact results. The resulting picture is expected to be closer to reality than that obtained using purely reported data, but worse than that correcting properly for age and diagnosis if the data of IFR for all age brackets and locations (nursing homes, hospitals, individual homes) were available, which is not the case.

The estimation of the diagnostic rate is straightforward. From the cumulative number of deceased each day, and multiplying by 100 (1% IFR), we get the cumulative number of people with symptoms 18 days ago [10–12] simply by rescaling and displacing the cumulative death curve of any country backwards in time. To give an initial realistic and homogenous diagnostic rate we must establish how many days are needed as a bare minimum to be able to detect a patient from the onset of symptoms. First, the patient has to feel sufficiently sick and then contact the health service. From this contact, the doctor needs to be suspicious that the person has the disease and request a test. Then, this test must be available, performed, and the result received and noted. It is clear that a bare minimum of one week is needed for this process. We use the term 7-Days Diagnostic Rate (7D-DR) for the diagnostic rate with a benchmark of one week of diagnosis delay.

**Fig 3:**
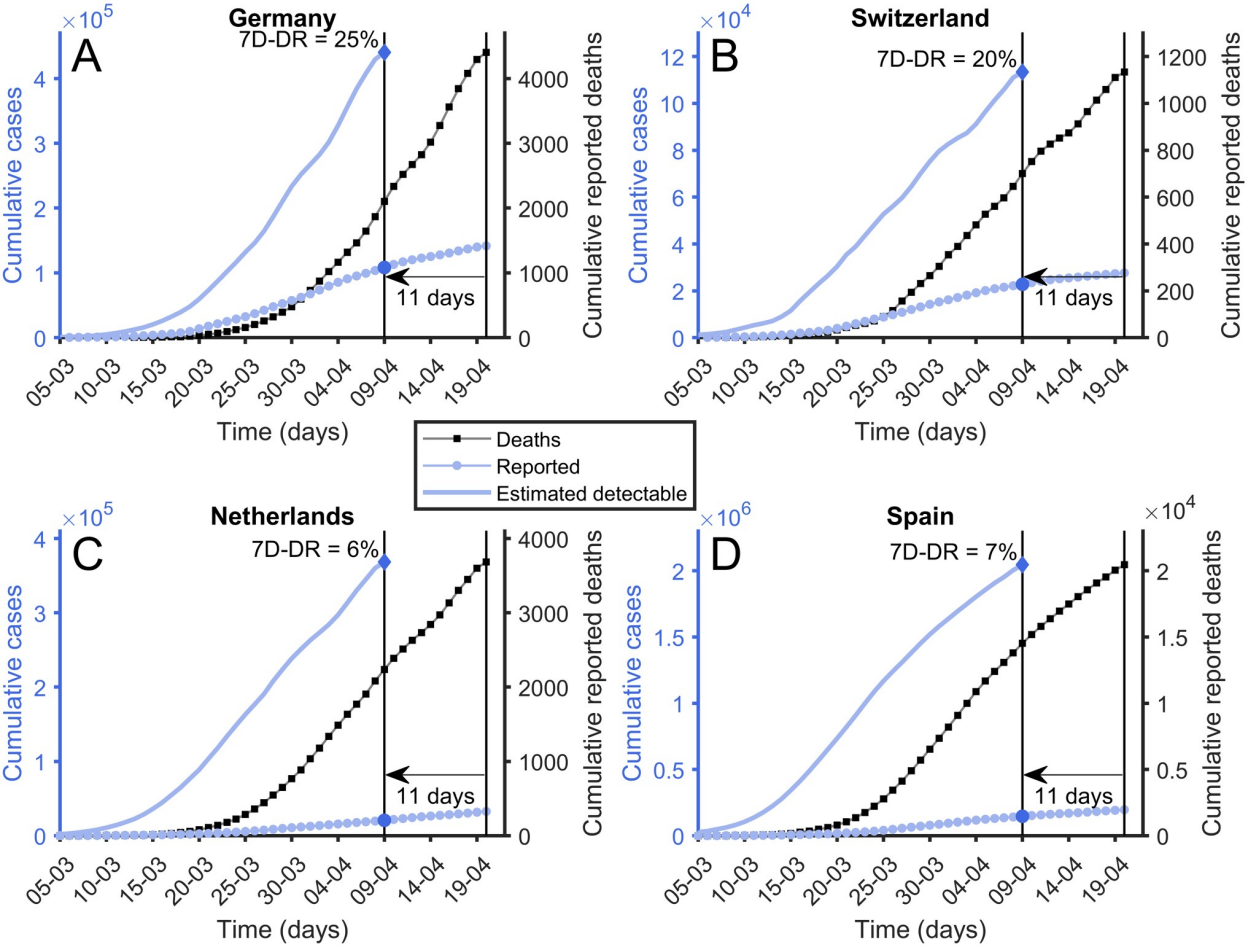
7-Days diagnostic rate. Reported cumulative number of deaths (black squares), reported cumulative number of cases (blue circles) and estimated number of cases calculated using Eq 1 (solid blue line). To compute 7-Days Diagnostic Rate a diagnosis-to-death time of 11 days is used. Its value is calculated using the latest available points. (A) Germany. (B) Switzerland. (C) Netherlands. (D) Spain.

We explain the procedure to get 7D-DR for each country in Fig 3A. We take the cumulative death curve of any country, rescale it according to the IFR, and displace it towards the past 11 days. This is 18 days back to the onset of symptoms and then 7 days forward to be detectable/diagnosable. From this curve, we can obtain the rate between the cumulative number of people who had symptoms for 7 or more days and the cumulated number of people detected 11 days ago. It is thus clear that this homogenous analysis across countries could be performed assuming 5D-DR or 9D-DR and different IFRs. It yields a proper first estimation of the situation.

**Fig 4:**
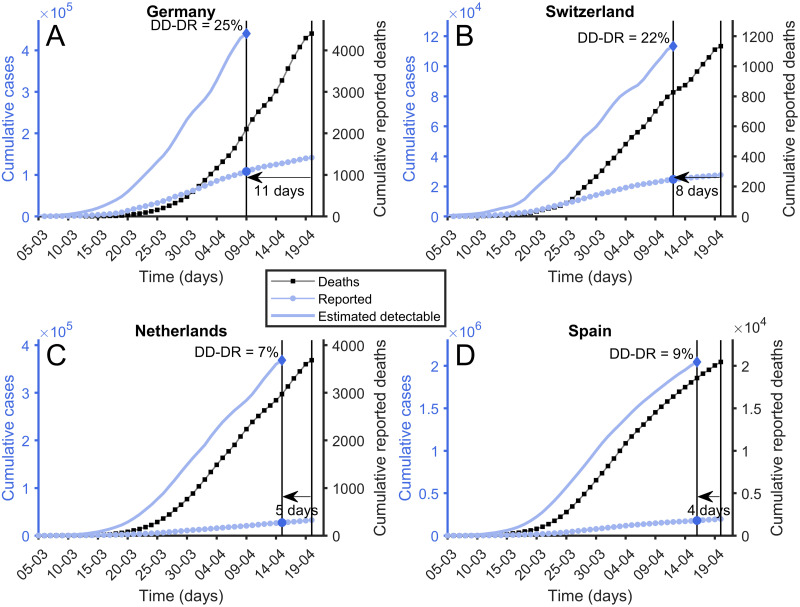
Delay-to-Detection Diagnostic Rate (DD-DR). Reported cumulative number of deaths (black squares), reported cumulative number of cases (blue diamonds), and estimated number of cases calculated using Eq 1 (solid blue line). To compute Delay-to-Detection Diagnostic Rate the diagnostic to death time observed in Fig 2 is used. Its value is calculated using the latest available points. (A) Germany, DtD = 10 days. (B) Switzerland, DtD = 8 days. (C) Netherlands, DtD = 5 days. (D) Spain, DtD = 4 days.

We argue, however, that there is indeed bias in the way people deal with the health care system in normal situations, and especially under epidemic circumstances. Different countries and populations in fact behave very differently. We have observed that this is the case in the Methods section checking the delay between diagnosis and death using time-displaced correlation analysis. This is the reason why we also define the Delay-to-Detection Diagnostic Rate (DD-DR) as the diagnostic rate computed using a different time delay between the appearance of symptoms and detectability for each country. We proceed to use Fig 4, with Spain as an example, to explain the concept behind DD-DR.

For Spain, the maximum correlation between cumulative death curves and cumulative reported cases appears when cumulative deaths are displaced 4 days backwards. This suggests a DD of around two weeks (18 − 4 = 14 days). This makes sense in a situation like that in Spain during March, 2020. The population receiving news that the health care system is under stress may decide to delay reporting of symptoms unless these are very serious. Additionally, there is the possibility that tests are not available to people who report to primary health care centers with symptoms, and that the delay between the test, its positive result, and its recording in official information systems is not negligible as well.

It is thus important to correct for this bias in the estimation of the diagnostic rate. It is clearly not the same to have a time delay from symptom to the detection of 14 days and 7 days. DD-DR can be computed from Spain just as we did before for the 7D-DR using the same rescaling of the cumulative death curve as before but using a displacement backwards of 4 days instead of 11 days. Fig 4 shows how the DD-DR is obtained in different countries depending on the delay between symptoms and detectability. Countries with a lower DD, such as Germany, have the same 7D-DR as DD precisely because they diagnose as early as realistically possible.

**Fig 5:**
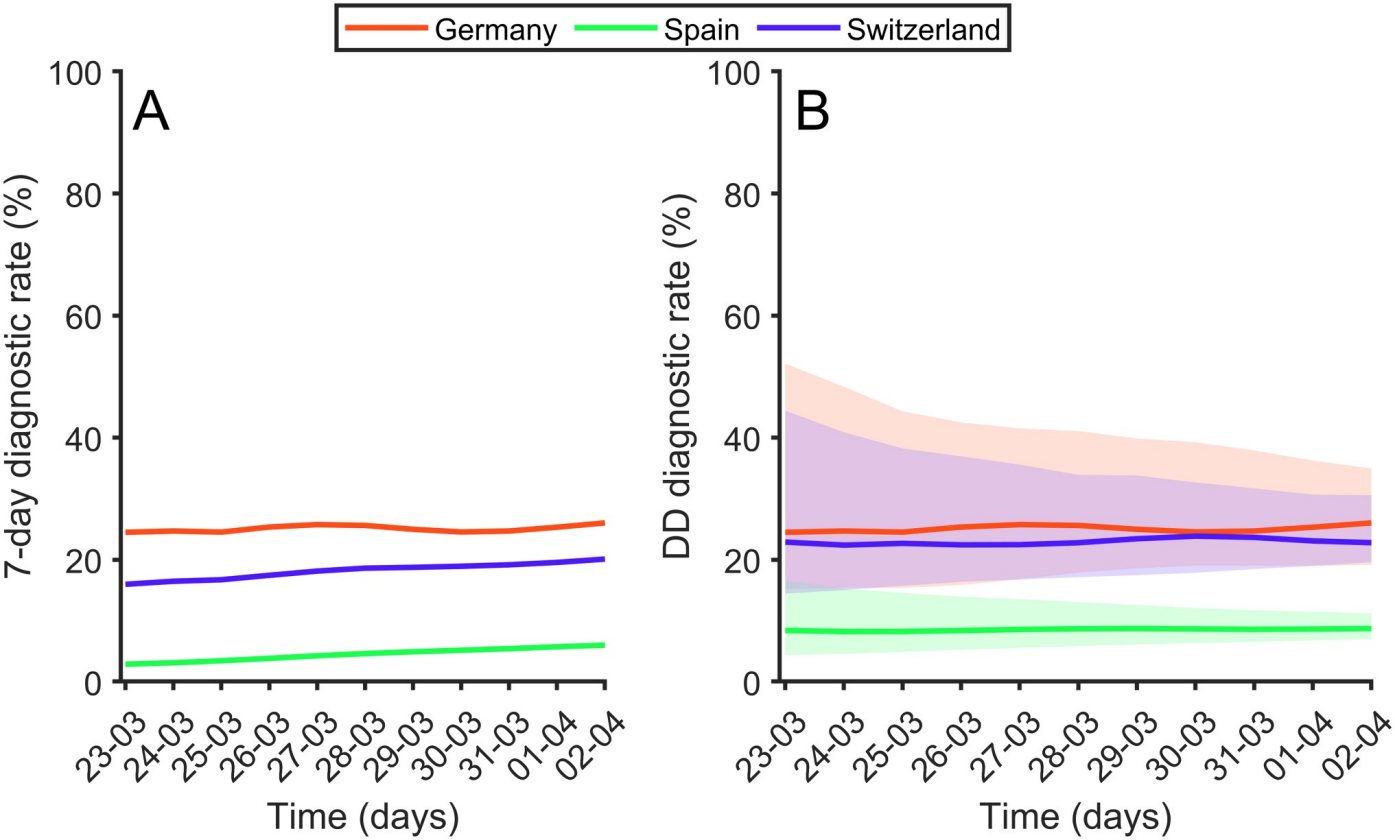
Diagnostic rate over time. (A) 7-Days Diagnostic rate over time for Germany (red), Spain (green), and Switzerland (blue). (B) Delay-to-Detection Diagnostic Rate over time. Thick lines are derived from the Diagnosis-to-Death time observed in Fig 2. Shaded areas represent the limits considering error bars observed in Fig 2.

We note now that both 7D-DR and DD-DR can be tracked over time; as the epidemic advances we can check how these diagnostic rates changes. Each new day we can look 11 days back for the 7D-DR and compute the diagnostic rate. DD-DR can be tracked similarly. In Fig 5 we show the evolution for both as a function of time for three selected countries. We observe that the DD-DR reaches a steady state after the initial stages of the disease while 7D-DR seems to be more affected by trends. This is to be expected since DD-DR uses, precisely, the maximum correlation delay and so it fluctuates less. The DD-DR is not only more stable but it also allows a proper assessment of the errors involved. The main error is the fact that the exact delay between onset of symptoms and detectability in each country shows large uncertainty. While the best estimation of the time delay in Spain is 14 days, the real value could be around 12. For Germany, for example, DD can be anywhere from just 4 days to 11 days. Using different time delays we obtain different diagnostic rates. It may be observed in Fig 5 that the percentages of diagnoses in Germany, and Switzerland are more or less constant over time. In the S1 File, we show the evolution of DD-DR for the 10 countries studied (see S1 Fig in S1 File). Assuming that this percentage remains constant to this day and that the diagnostic conditions have not changed over the last few days, we can estimate the total number of cases as of mid-April, 2020 to be roughly 2.3 million in Spain and close to a half million in Germany. We must notice that the ENE-COVID study [40] which surveyed the real incidence in Spain found that between April 27 and May 11, 2020 seroprevalence for the entire country was 5%, representing a total amount of people infected by COVID-19 of 2.4 million.

**Fig 6:**
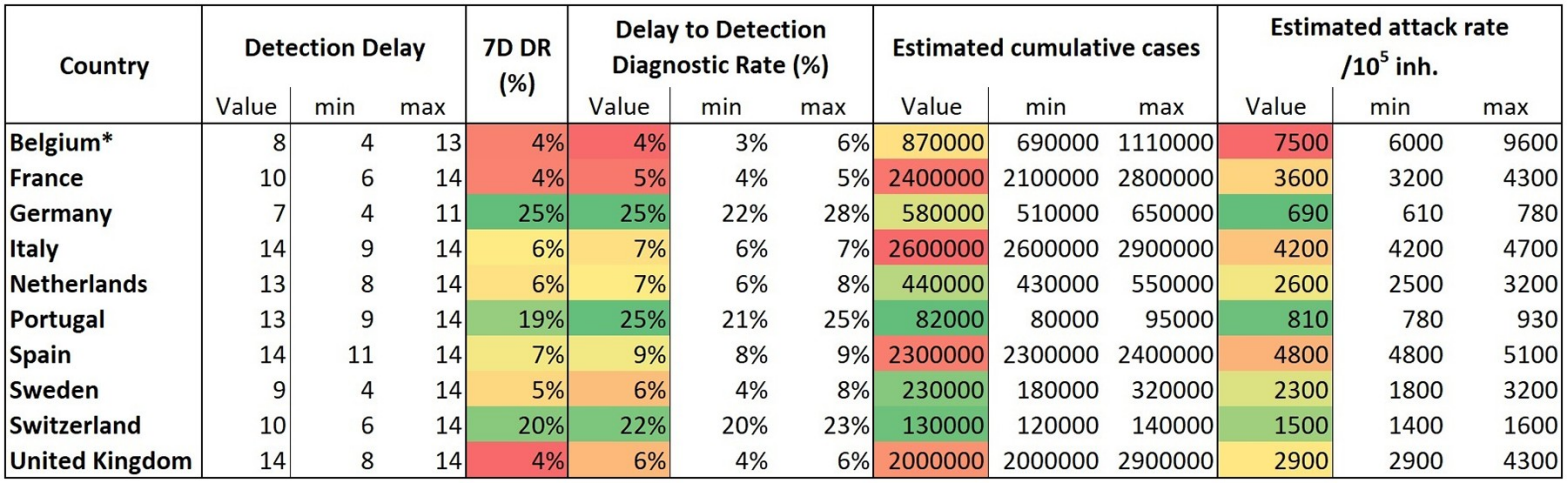
Detection Delay (DD), 7-Day Detection Rate (7D-DR), Delay-to-Detection Diagnostic Rate (DD-DR), estimated cumulative cases, and estimated attack rate. To interpret estimated cumulative cases and estimated attack rate we must take into account Detection Delay, because these are computed using the reported data. Data updated on April 20, 2020. Belgian data are biased due to reporting of unconfirmed death cases. On that date the shift was 48% [34].

The table in Fig 6 shows a list of the 7D-DR as of mid-April, 2020, and the DD-DR, which seems stable, together with the associated error.

### Effective Potential Growth (EPG) index for policy makers

Once the diagnostic rate is known, it is straightforward to establish a real incidence no longer affected by the presence of important differences in the time delays to diagnosis in different countries (see the table in Fig 6). The level of diagnosis and the real incidence is indeed useful for policymakers since it gives a clear general picture. However, the policy response needed to improve the diagnostic rate is conditioned, in the short-term, by the ability to increase the production of PCR kits and other diagnostic tools.

Policymakers have greater ability to immediately affect mobility patterns and social contact. In this sense, a key figure for policymakers would be to have a reliable and robust estimation of the number of infected people in each country that can propagate the disease. Providing an exact number is, right now, impossible.

We can, however, produce an index of the effective potential growth using the DD-DR and the guidelines used by the ECDC to track the epidemic. Even if the precise number of people with the disease were known, and the distribution of symptoms by sex and age was reported, there is no clear knowledge regarding the level of infectivity of the different types of person and symptom. For instance, it is not known for how many days an asymptomatic, pre-symptomatic or symptomatic person can transmit the disease [41–43]. Virus loads in the throat seem to be rather high across the board [44], but data on how this influences contagion is unclear. The only way to assess the situation is to use a general unbiased measure which is indicative of the potential for infection. The ECDC uses the number of newly infected people in the last 14 days [45]. We use this same criterion.

**Fig 7:**
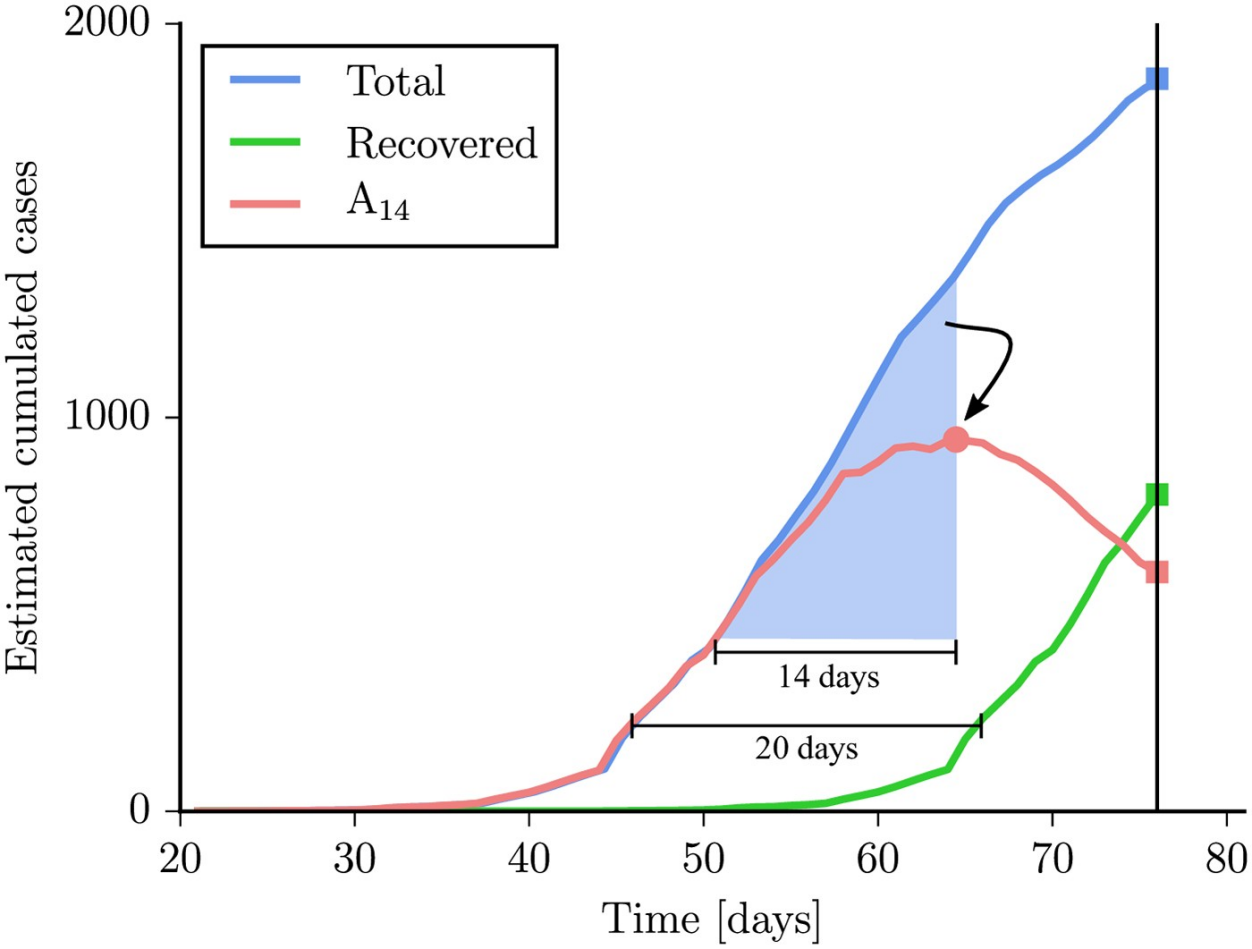
Schematics of the procedure to obtain incidence A_14_, recovered and estimated cases using Germany as an example. Incidence of estimated cases (blue), contagious incidence (red), and total estimated recovered cases (green). Blue shaded area is the number of cases used to compute the estimated contagious incidence. To interpret final number of total cumulative cases, recovered cumulative cases and estimated attack rate we must take into account Detection Delay, because they are computed using the reported data. Similar figures for all countries are shown in S2 Fig in S1 File.

Fig 7 shows how to compute an estimation of the people that go undetected and have the potential to transmit the disease. Using the DD-DR one can compute how many undetected people were added to the infected number in the last 14 days, I_14_. This number can only be obtained properly some days in the past, on the day that we have a typical diagnosis. After that, we would need input from new data to properly compute how many people are diagnosed. So the number I_14_ is strictly a measure of the recent past, but good enough to give a proper picture of what that the system will face in the following days.

We also consider those undetected cases which appear earlier than 14 days as recovered *R*_*I*_. Note that here we use the word recovered weakly. It does not mean literally that all of them are fully recovered since most of them never fell ill to begin with, and some of them could not have neutralized tests yet, but simply that those infected and undetected more than two weeks ago do not seem to pose a serious risk.

**Fig 8:**
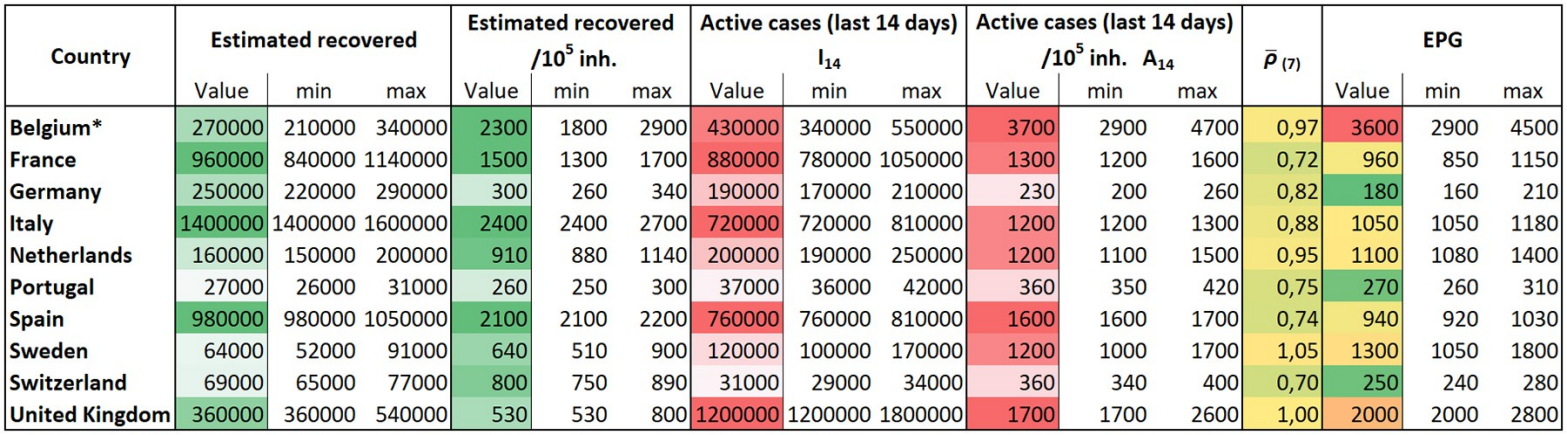
Estimated recovered and active cases, ${\bar{\rho }}_{\left(7\right)}$ and EPG. ${\bar{\rho }}_{\left(7\right)}$ is computed using the mean value for the last three days.EPG: Effective Potential Growth described in the text. To interpret table data we must take into account Detection Delay, because they are computed using the reported data. Data updated on April 20, 2020. *Belgian data are biased due to reporting of unconfirmed death cases. On that date the shift was 48% [34].

A list of values for I_14_ and the corresponding 14-day attack rate per 10^5^ inhabitants (A_14_) is provided for each country in the table of Fig 8 with the number computed at the beginning of April, 2020. These values are currently being monitored each day for all UE countries. Having an unbiased assessment of the risk regarding the number of potential spreaders, I_14_ and A_14_, is key to policymakers.

A_14_ alone, however, does not give a full picture of the situation. It is not the same to have 100 contagious per 10^5^ inhabitants when the number of contacts is high as when the number of contacts is low. It is important to take into account the level of spreading velocity of the epidemic related to the effective reproductive number (*R*_*t*_).

The effective reproductive number depends on multiple factors, from the properties of the virus itself to the number and types of contacts. Those, again, depend on different social behavior and structure such as mobility, density, and typical size of the family unit sharing a house, to name a few. The only feasible way to estimate *R*_*t*_ is using fits from Susceptible-Exposed-Infectious-Recovered (SEIR) models. Complex SEIR models which include spatial and contact-processes have a large number of parameters which, due to the present lack of knowledge, are unknown. This makes any estimation of *R*_*t*_ highly dependent on the value of other co-factors that strongly affect propagation. In essence, *R*_*t*_ can only be fit in very simple SEIR models where a small number of parameters are unknown and *R*_*t*_ can be calibrated from them.

Given the partially empirical nature of present *R*_*t*_, we prefer to take a fully empirical surrogate as a quantitative evaluation of the level of infections. We define an alternative reproductive number as the number of new cases detected today divided by the number of new cases detected five days ago: *N*_*t*_/*N*_*t*−5_. However, the high fluctuations of these quantities requires that we use averaged values [9]:

$$\begin{array}{c} \rho_{t}=\frac{\sum_{i=-n_{d}}^{n_{d}}N_{t+i}}{\sum_{i=-n_{d}}^{n_{d}}N_{t+\tau +i}}, \end{array}$$ (4)

where *N*_*t*_ stands for new cases reported at day *t*. Specifically we use values over three days (*n*_*d*_ = 1) and the delay *τ* = 5. We take 5 days as the key delay unit since this is roughly the time at which infected people develop symptoms if they do develop them.

$$\begin{array}{c} \rho_{t}=\frac{N_{t-1}+N_{t}+N_{t+1}}{N_{t-6}+N_{t-5}+N_{t-4}}. \end{array}$$ (5)

This rate is one if the number of new cases is constant. It will be below 1 if new cases are decreasing and larger than 1 if the number of cases is increasing.

There are still clear fluctuations on a day-to-day basis of this measure *ρ*_*t*_ due to common delay and irregularities in reporting. In April, 2020 there was also a slight weekend effect. Due to the reduction in working hours in the information systems and biotechnological firms, the number of new cases dropped during the weekends in every single country. The backlog of cases were normally declared the following days. This fact appears in the data reported until July 2020, and it has not diminished during the epidemic. Information systems are still not required to report on a day-to-day basis. A short average of three or four days should be enough to obtain an index which is representative fast enough to the situation. However, this weekend effect requires 7-day averages to get a proper picture. We define the average of *ρ*_*t*_ during seven days ${\bar{\rho }}_{\left(7\right)}$.

$$\begin{array}{c} {\bar{\rho }}_{\left(7\right)}=\frac{1}{7}\sum_{i=-3}^{3}\rho_{t+i}. \end{array}$$ (6)

We propose the following day-to-day EPG index:

$$\begin{array}{c} \text{EPG}={\bar{\rho }}_{\left(7\right)}A_{14}. \end{array}$$ (7)

EPG is just the multiplication of the growth rate of the disease ${\bar{\rho }}_{\left(7\right)}$ with the estimation of A_14_ both evaluated at the proper time in the recent past. The worst case scenario is one where both A_14_ and ${\bar{\rho }}_{\left(7\right)}$ are large. This means you had a large population with the disease and lots of spreading a few earlier. The best situation is a low value of velocity and low number of active cases. Having a large number of A_14_ with low ${\bar{\rho }}_{\left(7\right)}$ or a large ${\bar{\rho }}_{\left(7\right)}$ with low A_14_ are potentially dangerous situations. These values for the European countries we tracked on April 20, 2020, are given in the table of Fig 8. These values can be updated every day [9].

### Robustness of the EPG index

In this section, we check the robustness of the EPG index against the different parameters used. In order to compute the estimated EPG, we use the lethality of the virus (1%) and the delay between the onset of symptoms and death (TtD = 18 days). Both variables are estimated based on previous works [10, 13, 23, 24, 40]. The constants related to time delay in detection and from detection to death (DD and DtD) are calculated analysing the correlations and, consequently, they cannot be tuned. Similarly, the definition of active cases as those who have been detected in the last 14 days is used according to ECDC [32]. In order to smooth the effect due to the decrease of reported data during the weekends, a seven-days average value of *ρ*_*t*_ is used, ${\bar{\rho }}_{\left(7\right)}$.

However, there are two parameters that can be modified. First, in Eq (4), we assumed *τ* = 5 days. This is roughly the time since infected people develop symptoms if they do develop them. Given that there is a large uncertainty in this value, in the S1 File we study the sensitivity of ${\bar{\rho }}_{\left(7\right)}$ to changes in *τ* (3 and 7 days, see S1 Table in S1 File). No important differences are observed. The second parameter in Eq (4) is the number of days considered in the numerator and denominator, *n*_*d*_. We take it to be *n*_*d*_ = 1 but they could certainly be more. In order to test its robustness, we compare this value with the one using *n*_*d*_ = 3 days (one week) (see S1 Table in S1 File). It is straightforward to observe that the differences are small. The same analysis is presented there for the EPG index (see S2 Table in S1 File).

**Fig 9:**
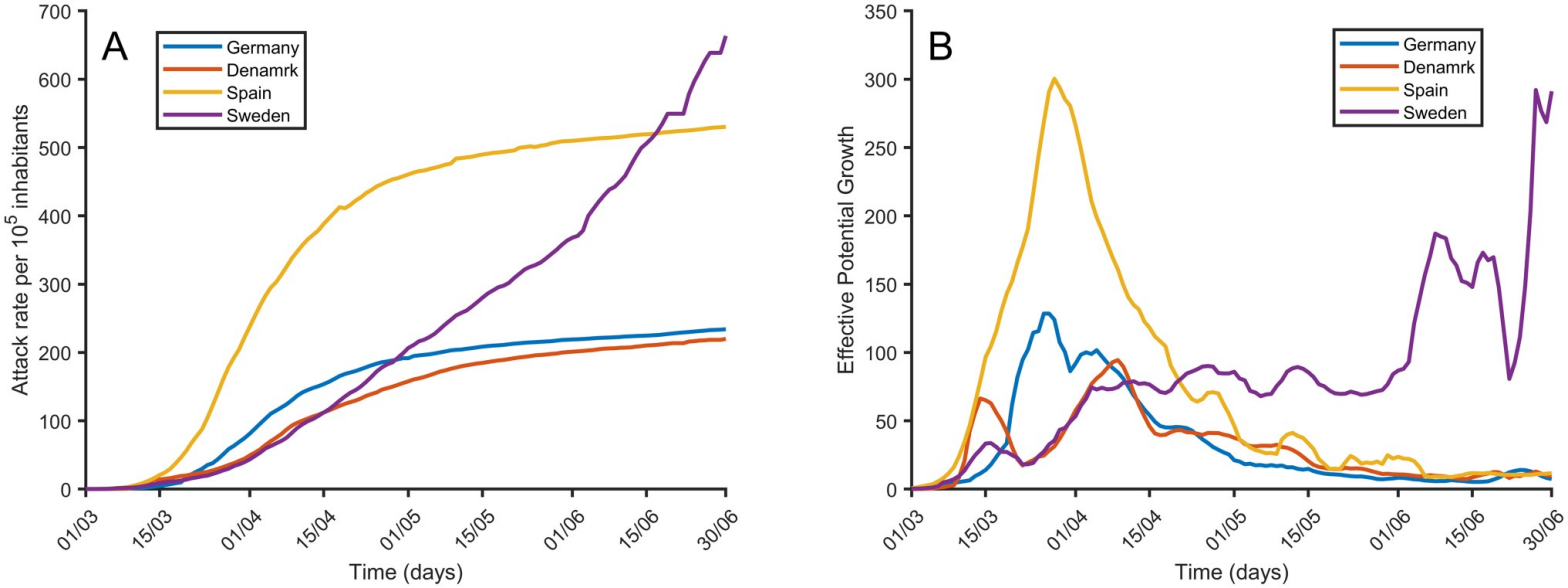
Attack rate and EPG for several countries. Temporal evolution of the attack rate per 10^5^ inhabitants (left) and the corresponding EPG (right) for four European countries with different epidemiological dynamics.

We must also address how robust the EPG has been in describing the epidemic so far. This was done by considering the evolution of four key European countries: Denmark, Germany, Spain, and Sweden. Fig 9 shows that the EPG behaved exactly as expected in the description of the different situations. The evolution of the epidemic in Denmark and Germany has been good and the EPG shows that this is the case. In Spain, a sharp increase in the attack rate followed by a rather fast decay is observed. The EPG (Fig 9B) increases earlier and consequently, if compared with the number of new cases, it is able to advance the troublesome situation in Spain. After the peak, it properly shows a decay showing a situation under control. On the other hand, Sweden has followed a trajectory of different stages without a sharp increase and later decay. During the later weeks depicted in Fig 9, we may observe an increase in EPG, which has been followed by an increase in cases, as expected.

**Fig 10:**
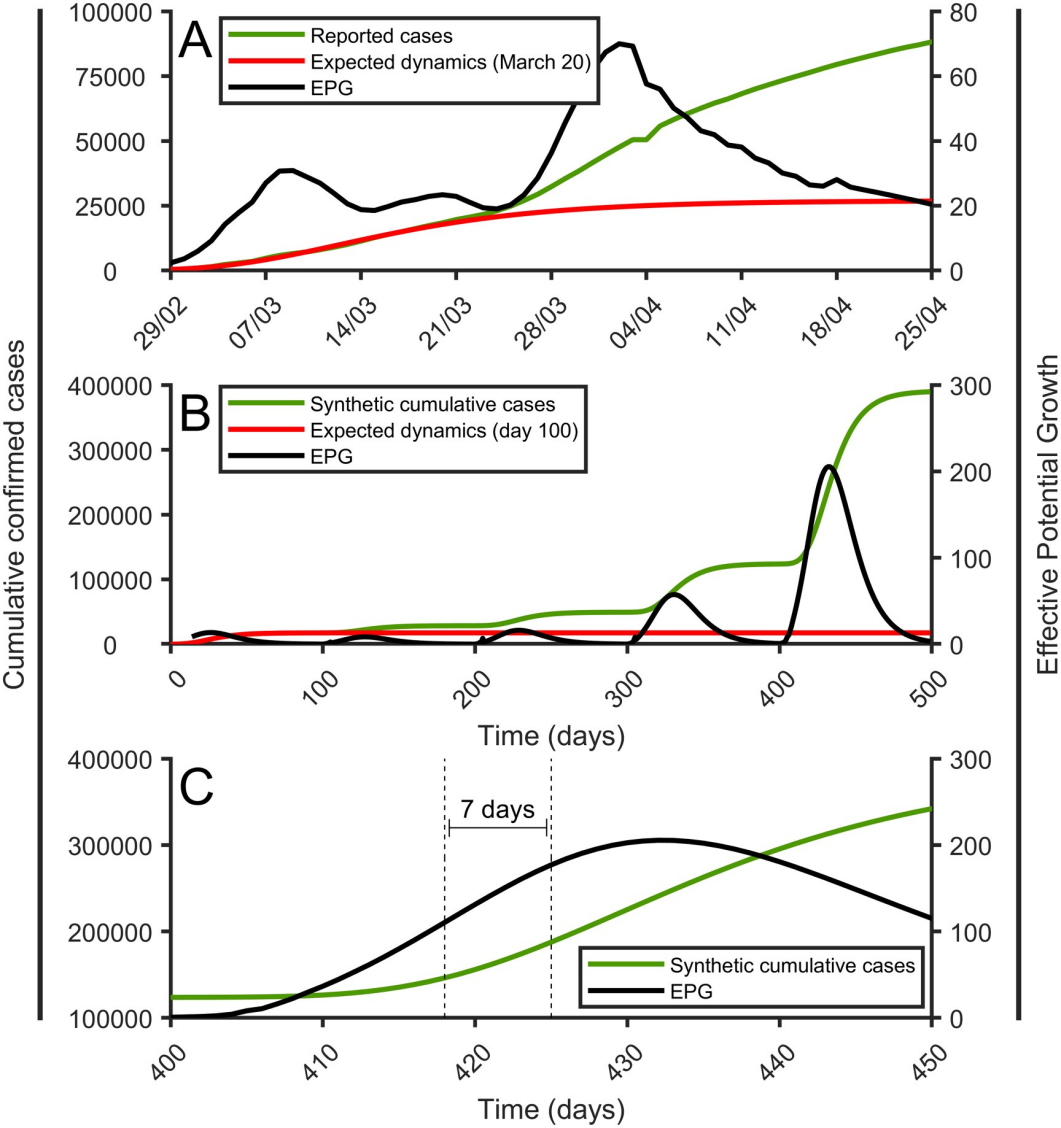
Evolution of the cumulative cases and the EPG. Evolution of the cumulative cases and the EPG. (A) The reported cases in The Republic of Iran (green line) is shown in comparison with the expected dynamics (red line) and with the calculated EPG (black line). (B) Same for the synthetic data. (C) Zoom of B panel between days 400 and 450.

We have shown that an increase of the EPG indicates correctly that the incidence during the first phase of the epidemic will increase during the following days. Additionally, it would be interesting to also analyze if the evolution of the EPG can be used as a proxy to predict secondary outbreaks once the first wave of the epidemic has abated, and what is the delay between the beginning of the outbreak and the EPG-response. European countries, as of mid-July 2020, do not present these secondary outbreaks. We need to look for a good example outside of Europe. We think the perfect example to analyze secondary outbreaks is Iran. The country has suffered a secondary and, probably, a tertiary outbreak as indicate the evolution of the number of cumulative cases shown in Fig 10A. The figure also shows that the EPG index properly predicts the new outbreaks in Iran.

However, the only way to provide quantitative values for the delay between the beginning of the outbreak and the EPG-response is to use surrogate data which properly mimics the evolution of the epidemic. In surrogate data, we can control precisely the exact moment of the initiation of the outbreak. To this end, we use Gompertz-like generated data. The evolution of the epidemic in all the countries of the world and in the different regions and states can be properly fitted by a Gompertz function [46]. Primary and secondary outbreaks can be properly fit by consecutive Gompertz functions, as shown in Fig 10B. In order to assess this delay, we simulate four different outbreaks using Gompertz surrogate structures providing different strengths for them. The expression of the cumulative cases, *C*(*t*), as a function of the day *t* in each one of the surrogate outbreaks reads:

$$\begin{array}{c} C\left(t\right)=Ke^{-\ln\left(\frac{K}{N_{0}}\right)e^{-a(t-t_{0})}}. \end{array}$$ (8)

The different surrogate outbreaks have different parameters *K* and *t*_0_, being the final number of cases of the secondary outbreak and the time where we introduce it, respectively. Fig 10B shows the four surrogate consecutive outbreaks with increasing magnitude and the associated EPG. In all of them, the EPG raises and detects the increase. If the new outbreak is small the EPG never crosses the threshold value of 100, indicating that, for the secondary outbreak, a low value of *K* does not produce significant increases in EPG. As long as the increase is large, EPG reaches values above 50 very rapidly. In Fig 10C we can observe the delay between the starting day of the surrogate outbreak *t*_0_ and the peak in the evolution of the EPG. We can see how the outbreak peaks just two weeks after the outbreak. If we focus on its rapid initial increase, the outbreak can be detected clearly within 5-10 days of its onset. This is very early considering the time needed for the epidemic to develop, with typical times scales of infection around five days or one week. This surrogate analysis shows how robust is the EPG index in detecting relevant secondary outbreaks.

## Discussion

The reported number of deaths per 100,000 people is a fairly objective and relatively simple way of assessing the situation of the COVID-19 epidemic in the different countries. The complete picture must be given by a more complex analysis of other data such as the number of diagnoses per 100,000 inhabitants, distribution of these cases among regions and according to age and sex, percentages of asymptomatic and mild cases, and spreading rate of the epidemic, among others. Nevertheless, any analysis based on diagnosed cases is biased by diagnosis protocols and ratios in each country, as well as by the pool of asymptomatic cases. Moreover, any attempt to improve diagnostic percentage requires an economic, infrastructural, and logistical effort that is not always possible. In addition, this health system structure imposes a strong condition that limits the possible actions to carry out in this direction. The reported number of deaths, if uniformly and properly recorded, provides very relevant information as a first general overview. Even in countries where there is a bias in reporting death, the effort that should be made to improve these data collection is much lower than the effort needed to increase data about cases.

The assumption of a common IFR, which has been situated around 1%, allows for using the IFR as an indicator of the real incidence. Current information on IFR is still not complete, since many countries do not report distribution of death by age or sex, nor information on COVID-19 mortality outside hospitals. However, we argue that the picture that we obtain from the analysis using IFR is closer to reality than the one provided by the pure analysis of reported cases. In particular, this analysis allows for: (1) establishing an order of magnitude of real cases and diagnostic percentage, (2) assessing an effective potential growth index to evaluate the risk, and (3) obtaining an order of magnitude of recovered people that could be potentially immunized in the short-term.

In Europe, absolute case ranking has been lead by Italy (until April 4, 2020) and Spain (since then). On April 20, 2020, Spain was at the level of 196,000 reported cases while Italy was reporting 179,000. They were followed by Germany (142,000), the United Kingdom (120,000), France (113,000), and Belgium (38,000). If we estimate the cases that should have been diagnosed by that time, the ranking is led by Italy (2,600,000) and followed by France (2,400,000), Spain (2,300,000), the United Kingdom (2,000,000), Belgium* [33] (870,000), and Germany (580,000). Thus, differences in diagnostic rate are absolutely significant when analyzing the global situation in Europe. In April 2020, countries like Germany, Portugal, and Switzerland would be diagnosing around 25% of cases, while Belgium, France, Sweden, and the United Kingdom would be at the level of 5%.

Assessing the risk of countries to enter or remain in the epidemic growth phase is essential. In this sense, the EPG index is a valuable tool for policy makers. A high EPG in the situation where there is a high growth rate of the epidemic and a large number of active cases is a clear situation of danger, while a very low EPG because both the number of recently infected and the spread velocity are low is clearly a controlled situation. In intermediate situations, EPG informs whether the growth rate is too high for the number of infected at hand. Even if ${\bar{\rho }}_{\left(7\right)}<1$ and the epidemic seems to be under control because new cases are decreasing, intermediate EPG values informs the policy maker that reopening can have a very important cost in the form of secondary outbreaks and waves of infection. A rather large EPG with low ${\bar{\rho }}_{\left(7\right)}$ is a situation in which the number of spreaders is potentially very high and increasing the number of contacts carries a large risk. Therefore, EPG is a very informative index that is also very robust.

**Fig 11:**
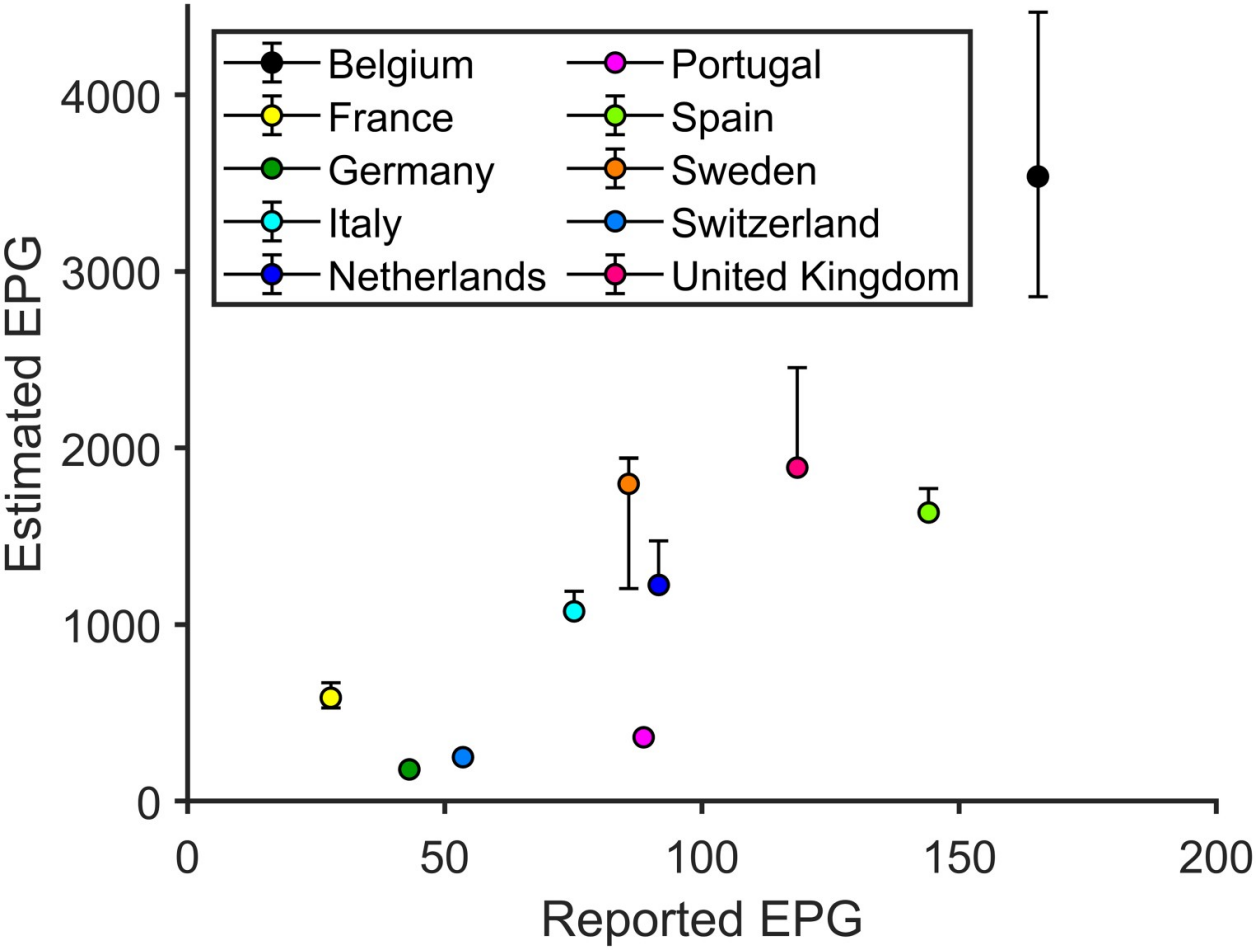
Reported EPG vs estimated real EPG. Several European countries in terms of the EPG computed using the reported data on the attack rate vs the EPG using our estimation of the real attack rate. The order of the different countries should be done from right to left (for the reported state of the index) and from top to bottom (for the estimated value of the index). We observe how the comparative situation of the different countries changes as of April 20, 2020. * Belgian data are biased due to reporting of unconfirmed death cases. On that date the shift was 48% [34].

Although ${\bar{\rho }}_{\left(7\right)}$ is independent of the diagnostic rate, reported I_14_ directly depends on the level of diagnosis. Thus, if EPG is evaluated by using the reported data (reported EPG), it can provide an erroneous picture of the situation. Based on reported EPG, the worst situation in Europe on April 20, 2020 would be that of Belgium, followed by Spain, the United Kingdom, the Netherlands and Portugal. If risk is evaluated with the EPG calculated using the estimated cases (estimated EPG), the highest value would still correspond to Belgium, but followed by Sweden, the United Kingdom, Spain, the Netherlands, and Italy. Portugal is in a much better position than its reported data suggest. Actually, countries with similar reported EPG like that of Portugal, and Netherlands have, in fact, a totally different estimated EPG, with the last country at significantly higher risk than the other nine. Fig 11 shows visually how reported and estimated EPG compare.

We have shown in the Methods section that the basis for obtaining estimated I_14_ and A_14_ is not biased due to demographic differences and, at present, there is no indication that it is biased due to a different way of accounting for the cumulative death toll of the epidemic. There is also no indication that comorbidity factors are significantly different in different countries or that IFR is higher in some countries given that ICU units and hospitals are not available for people that might need them, at least to date. If this were the case, under any scenario where the situation occurs, the epidemic in that country would have a number of cases, attack rate, and growth so much greater and the EPG would be extremely high. The only real limitation is that the social and environmental issues could affect the prognosis of the infected. If living in a small house with other people infected could lead to worse prognosis than staying in a large house alone, a new analysis regarding the unbiased nature of the IFR would need to be made.

It is important to indicate that not only I_14_ is unbiased, as noted in the previous sections, but also that ${\bar{\rho }}_{\left(7\right)}$ is unbiased as well. Even though the absolute number of reported cases is biased, as we have shown (Eqs 4 and 5), ${\bar{\rho }}_{\left(7\right)}$ deals with ratios and its evolution. As long as the diagnosis and recording of the people with disease follows roughly the same criteria over time in each country, ${\bar{\rho }}_{\left(7\right)}$ is a good measure of the growth of the epidemic. Indeed, if evaluated diagnosis percentage is more or less constant over time, we can assume that ${\bar{\rho }}_{\left(7\right)}$ correctly reveals tendencies in contagiousness. If a change in criteria in reporting the cases occurs (i. e., a large increase in the number of tests per day leading to an increase in cases due to more testing), ${\bar{\rho }}_{\left(7\right)}$ will be temporally affected but will re-establish itself as a good measure once the new criteria are established. In this case, EPG will provide an erroneous picture for a while as well, until stable conditions in diagnosing and reporting are again achieved.

There is another important point to address in order to guarantee that ${\bar{\rho }}_{\left(7\right)}$ is a robust measure. As long as we are estimating real number of cases, we can determine the associated ${\bar{\rho }}_{\left(7\right)}$. It is expected that the reported and the estimated ${\bar{\rho }}_{\left(7\right)}$ will behave similarly but with a certain delay. This delay can be determined by translating both ${\bar{\rho }}_{\left(7\right)}$ in time until the error between the two is minimized. We show this analysis in the S1 File, where we describe how the reported ${\bar{\rho }}_{\left(7\right)}$ and the estimated ${\bar{\rho }}_{\left(7\right)}$ are indeed different, but that both follow the same type of evolution once the proper delay is accounted for (see S3 Fig in S1 File).

The third important outcome of this analysis is the estimation of the number of people recovered. This is an important number in examining the possibility of herd immunity discussed as a possible exit strategy. The idea is that those that recover might have immunity and act as barriers in the transmission of the disease. A recent study by the Fudan University in Shanghai [32] analyzed antibody titers of 175 adult COVID-19 recovered patients. The study was based in the detection in plasma of Spike-binding antibody using RBD, S1, and S2 proteins of SARS-CoV-2 using an ELISA technique. It was also the first study to look at neutralizing antibodies (NAbs) specific for SARS-CoV-2 using a gold standard to evaluate the efficacy of vaccines against smallpox, polio, and influenza viruses. The study highlights the correlation between the NAb titers and Spike-binding antibodies that were detected in patients from day 10-15 after the onset of the disease, and which remained afterwards. Middle and elderly age patients had higher titers compared to young age patients, among whom in 10 cases the titers were under the limit of detection. NAb titers had a positive and negative correlation with C-reactive protein (CRP) levels and lymphocyte counts, respectively. This indicates that the severity of the disease, in terms of inflammatory response (CRP levels), is usually worse in the middle-aged and elderly, and favors the increase of antibody titers. Equally, the negative correlation with lymphocyte counts suggests an association between cellular and humoral response. Therefore, it is possible that the immunity achieved by young people, who were mostly asymptomatic, is residual. In that case, this sub-population would continue being carriers of COVID-19. Recent serological studies [40, 47, 48] have shown that there is some post-infection immunity, but it isn’t clear how long this immunity will last [49, 50]. Besides antibody immunity, T-cell immunity might play an important role [51, 52] and, additionally, some persons might have pre-existing immunity to SARS-CoV-2 [53].

Regarding the possibility of using herd-immunity as strategy for easing lockdown, the serological studies mentioned above have shown that, even the areas with the largest incidence of the pandemic are far from the necessary percentage of cases among their population to achieve herd immunity. In Europe the largest proportion of positive samples in a serological study was obtained in the United Kingdom with 8.5% of the population (see [54] and the references included there).

### Limitations

There are two possible limitations of the present study. It is possible, in theory, that some countries present an intrinsically different IFR if they are able to significantly isolate their elderly population more than others [55, 56]. The IFR is a measure of the case fatalities if all the population, or a representative sample of it, has become infected. If one country effectively prevents all infections among all its elderly population forever, the age group with the largest mortality, it will certainly have a different IFR, probably much lower. Right now, it is impossible to determine whether this is indeed the case in different countries given the lack of reported cases and case fatality ratios by age and sex. We should note, however, that if this disaggregation were to be provided, we could proceed with exactly the same methodology but instead of using the country as a whole we would divide it into different age brackets and treat them separately.

The second limitation is related to the first one but comes from a more structural perspective. A clear possibility is that countries under stress could be failing to provide the same medical support, and consequently increasing the IFR. We note that the health care systems in European countries, even under stress, have been able to dramatically increase the numbers of personnel, beds and hospitalization volume on short notice [57, 58]. Italy and Spain have had some regions under stress but not the whole country [59]. Finally, one cannot rule out the possibility that complex mechanisms of mutations and repeated exposure to the virus may change the prognosis. Therefore, the type of housing, and hence the socio-economic factors, which are clearly different from country to country, might influence the mortality if there were proof that a close environment not only increased the level of infection, which it obviously does, but also changed the disease evolution in the patient. In this case, one would again need to test whether the uniform/unbiased IFR hypothesis held with the knowledge at hand.

## Conclusion

We have estimated the diagnostic rate of European countries in an unbiased way and reported EPG (Effective Potential Growth) as an effective index to monitor the comparative situation of COVID-19 in different European countries. The diagnostic rate is different in each country but roughly constant over time. In addition, EPG changes for each country and at each stage of the epidemic its becoming large would signal a worrying situation.

## Supporting information

S1 FileSupporting information file.This includes two tables showing the sensitivity of ${\bar{\rho }}_{\left(t\right)}$ and EPG to different values *τ* and *n*_*d*_. The first figures show the correlation to obtain DtD for each country and the corresponding evolution of the diagnostic rate. We also provide the evolution of recovered and the attack rate in the last 14 days A_14_ for each country. We provide a demonstration that ${\bar{\rho }}_{\left(7\right)}$ is also unbiased showing the correlations between real and estimated growth rates.(PDF)
