# Supplementary material for: Robust estimation of diagnostic rate and real incidence of COVID-19 for European policymakers
Source: PLoS One. 2021 Jan 7;16(1):e0243701. doi: 10.1371/journal.pone.0243701 (PMC7790392; doi:10.1371/journal.pone.0243701)
Supplement: S1 File — This includes two tables showing the sensitivity of ρ¯(t) and EPG to different values τ and nd. The first figures show the correlation to obtain DtD for each country and the corresponding evolution of the diagnostic rate. We also provide the evolution of recovered and the attack rate in the last 14 days A14 for each country. We provide a demonstration that ρ¯(7) is also unbiased showing the correlations between real and estimated growth rates. (PDF) [file pone.0243701.s001.pdf]

S1 File

M. Català[1,2], D. Pino[1], M. Marchena[1], P. Palacios[1], T. Urdiales[1], P.J. Cardona[2,3,4], S. Alonso[1], D. López-Codina[1], C. Prats[1,2], and E. Alvarez-Lacalle[1]

[1] *Departament de Física. Universitat Politècnica de Catalunya-BarcelonaTech. Barcelona. Spain.*
[2] *Comparative Medicine and Bioimage Centre of Catalonia (CMCiB), Fundació Institut d'Investigació en Ciències de la Salut Germans Trias i Pujol, Badalona, Catalonia, Spain.*
[3] *Experimental Tuberculosis Unit (UTE). Fundació Institut Germans Trias i Pujol (IGTP). Universitat Autònoma de Barcelona (UAB). Edifici Mar. Can Ruti Campus. Crtra. de Can Ruti, Camí de les Escoles, s/n, 08916, Badalona, Catalonia, Spain.*
[4] *Centro de Investigación Biomédica en Red de Enfermedades Respiratorias (CIBERES). Av. Monforte de Lemos, 3-5. Pabellón 11. Planta 0. 28029, Madrid, Spain.*

Contents

In this supplemental material, we provide supplemental information about four features:

- We provide two tables showing the sensitivity of $\bar{\rho}_{(7)}$ and EPG to changes in the parameters τ, n_d (see Eqs. 4-7).
- We show the correlation to obtain DtD for each country and the corresponding evolution of the diagnostic rate.
- We show for each country the evolution of people recovered from the infection and the attack rate in the last 14 days, A_{14}.
- We show the correlations between $\bar{\rho}_{(7)}$ (see Eq. 6) computed from the reported data and from the estimated real data.

S1 Table : $\bar{\rho}_{(7)}$ (Eq. 6) for different values of the parameters τ, n_d (see Eqs. 4 and 5). In bold is marked the data used for the report. Data updated on April 20, 2020 from[1].

Country	$n_d = 1$			$n_d = 3$		
	$\tau = 3$	$\tau = 5$	$\tau = 7$	$\tau = 3$	$\tau = 5$	$\tau = 7$
Belgium	0,98	**0,97**	0,90	0,97	0,98	0,99
France	0,79	**0,72**	0,45	0,87	0,84	0,81
Germany	0,94	**0,82**	0,76	0,84	0,73	0,66
Italy	0,93	**0,88**	0,82	0,94	0,91	0,88
Netherlands	0,98	**0,95**	0,88	0,98	1,00	1,03
Portugal	0,82	**0,75**	0,53	0,85	0,85	0,85
Spain	0,83	**0,74**	0,59	0,84	0,76	0,69
Sweden	1,08	**1,05**	1,12	1,01	1,00	1,04
Switzerland	0,81	**0,70**	0,55	0,78	0,68	0,59
United Kingdom	0,98	**1,00**	0,91	1,02	1,08	1,16

S2 Table : EPG (Eq. 7) for different values of the parameters τ, n_d (see Eqs. 4 and 5). In bold is marked the data used for the report. Data updated on April 20, 2020 from[1].

Country	$n_d = 1$			$n_d = 3$		
	$\tau = 3$	$\tau = 5$	$\tau = 7$	$\tau = 3$	$\tau = 5$	$\tau = 7$
Belgium*	3633	**3580**	3321	3596	3642	3667
France	1064	**965**	605	1169	1131	1093
Germany	213	**184**	171	188	164	148
Italy	1112	**1054**	975	1116	1081	1045
Netherlands	1142	**1110**	1033	1141	1168	1203
Portugal	294	**269**	192	304	307	307
Spain	1064	**944**	757	1080	978	885
Sweden	1817	**1768**	1884	1696	1677	1755
Switzerland	291	**250**	197	279	243	212
United Kingdom	1969	**2023**	1834	2050	2172	2327

S1 Fig: Diagnostic rate and its evolution for each country

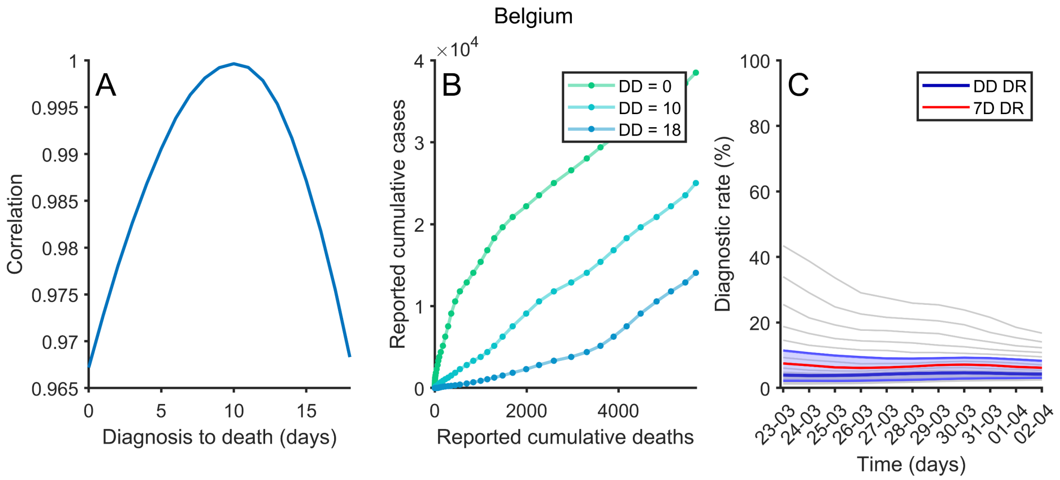

(A) Correlation between reported number of cumulative cases and reported number of cumulative deaths using different DtD times. **(B)** Alignment between reported number of cumulative cases and reported number of cumulative deaths using three different detection delays (DD). **(C)** Diagnostic rate along time using different DD, from top to bottom 0 to 18 days. In red, 7-day detection rate and, in blue, Delay to Detection Diagnostic Rate.

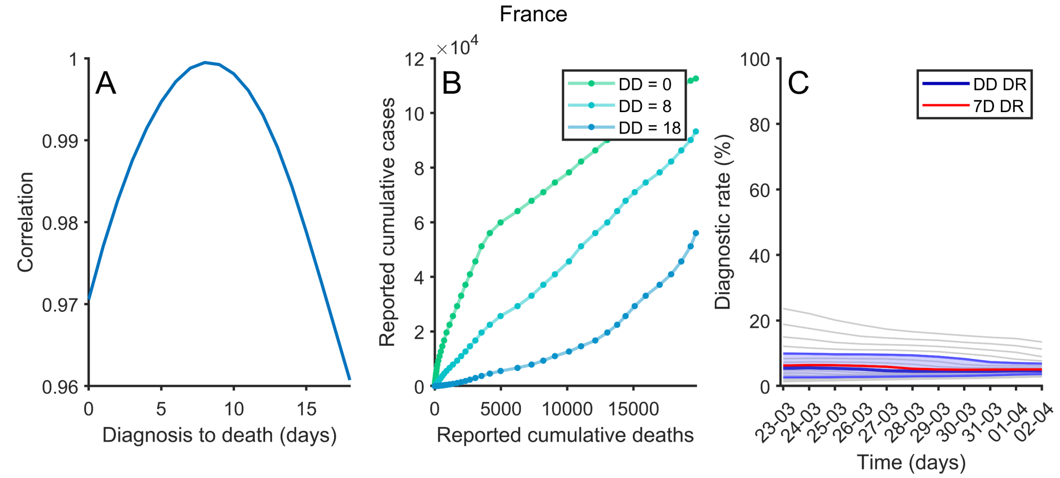

(A) Correlation between reported number of cumulative cases and reported number of cumulative deaths using different DtD times. **(B)** Alignment between reported number of cumulative cases and reported number of cumulative deaths using three different detection delays (DD). **(C)** Diagnostic rate along time using different DD, from top to bottom 0 to 18 days. In red, 7-day detection rate and, in blue, Delay to Detection Diagnostic Rate.

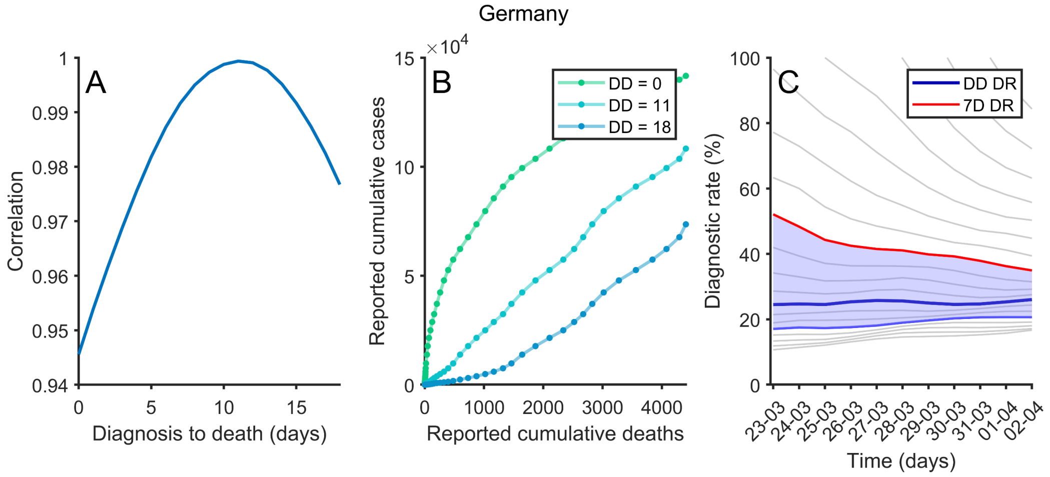

(A) Correlation between reported number of cumulative cases and reported number of cumulative deaths using different DtD times. **(B)** Alignment between reported number of cumulative cases and reported number of cumulative deaths using three different detection delays (DD). **(C)** Diagnostic rate along time using different DD, from top to bottom 0 to 18 days. In red, 7-day detection rate and, in blue, Delay to Detection Diagnostic Rate.

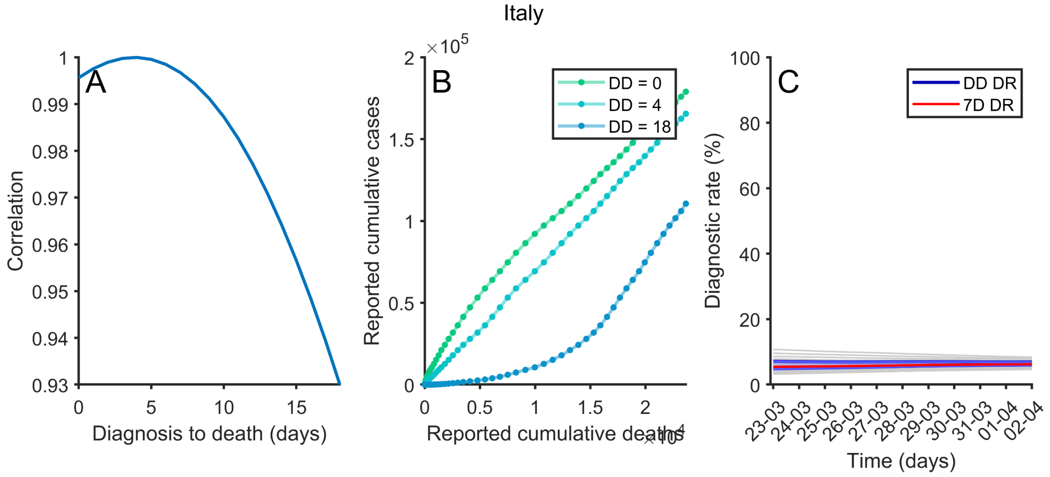

(A) Correlation between reported number of cumulative cases and reported number of cumulative deaths using different DtD times. **(B)** Alignment between reported number of cumulative cases and reported number of cumulative deaths using three different detection delays (DD). **(C)** Diagnostic rate along time using different DD, from top to bottom 0 to 18 days. In red, 7-day detection rate and, in blue, Delay to Detection Diagnostic Rate.

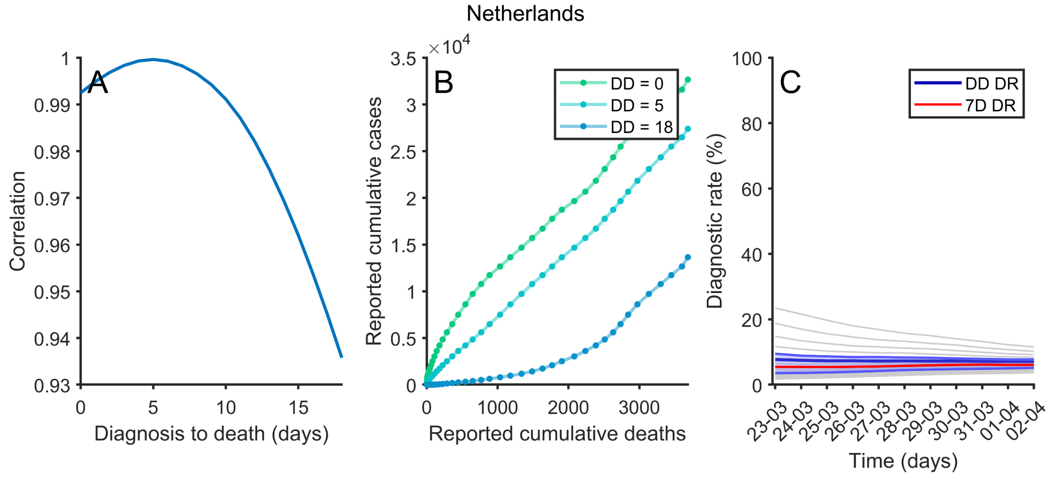

(A) Correlation between reported number of cumulative cases and reported number of cumulative deaths using different DtD times. **(B)** Alignment between reported number of cumulative cases and reported number of cumulative deaths using three different detection delays (DD). **(C)** Diagnostic rate along time using different DD, from top to bottom 0 to 18 days. In red, 7-day detection rate and, in blue, Delay to Detection Diagnostic Rate.

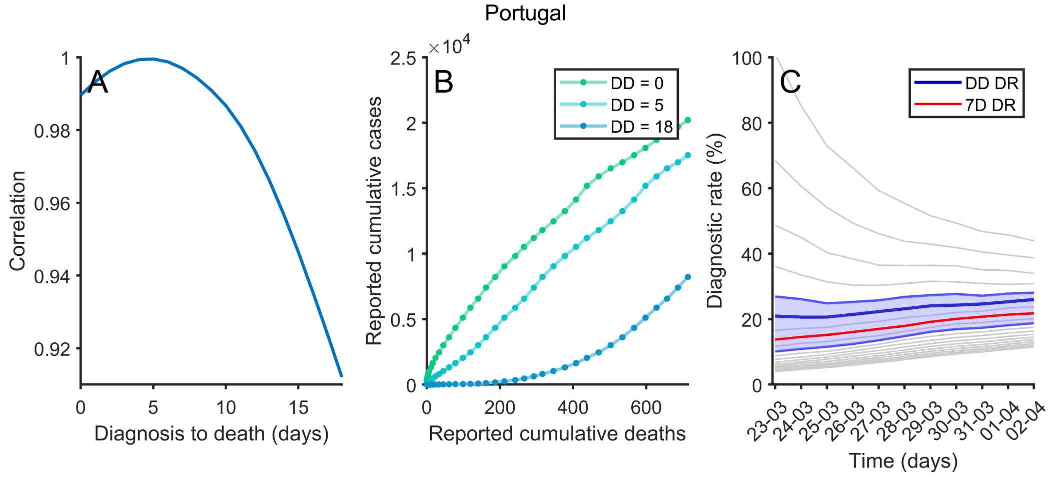

(A) Correlation between reported number of cumulative cases and reported number of cumulative deaths using different DtD times. **(B)** Alignment between reported number of cumulative cases and reported number of cumulative deaths using three different detection delays (DD). **(C)** Diagnostic rate along time using different DD, from top to bottom 0 to 18 days. In red, 7-day detection rate and, in blue, Delay to Detection Diagnostic Rate.

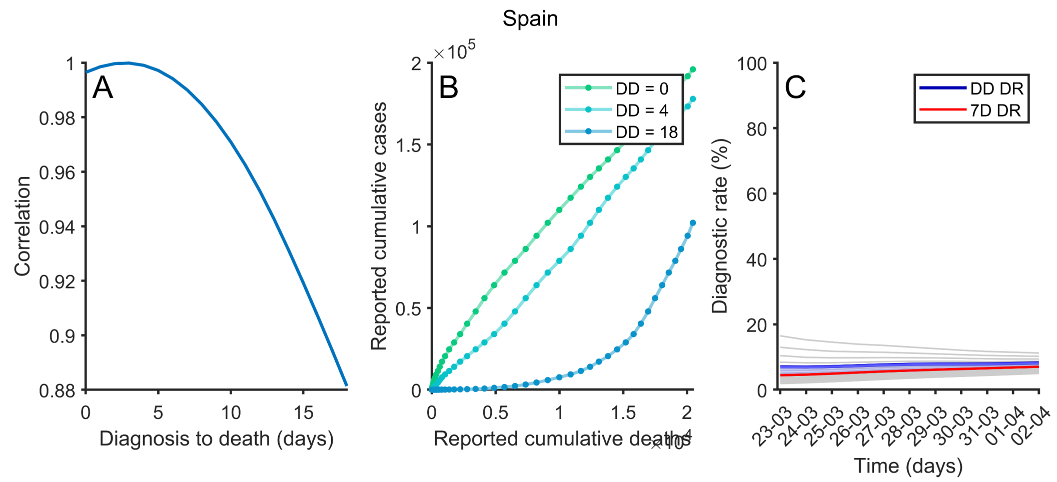

(A) Correlation between reported number of cumulative cases and reported number of cumulative deaths using different DtD times. **(B)** Alignment between reported number of cumulative cases and reported number of cumulative deaths using three different detection delays (DD). **(C)** Diagnostic rate along time using different DD, from top to bottom 0 to 18 days. In red, 7-day detection rate and, in blue, Delay to Detection Diagnostic Rate.

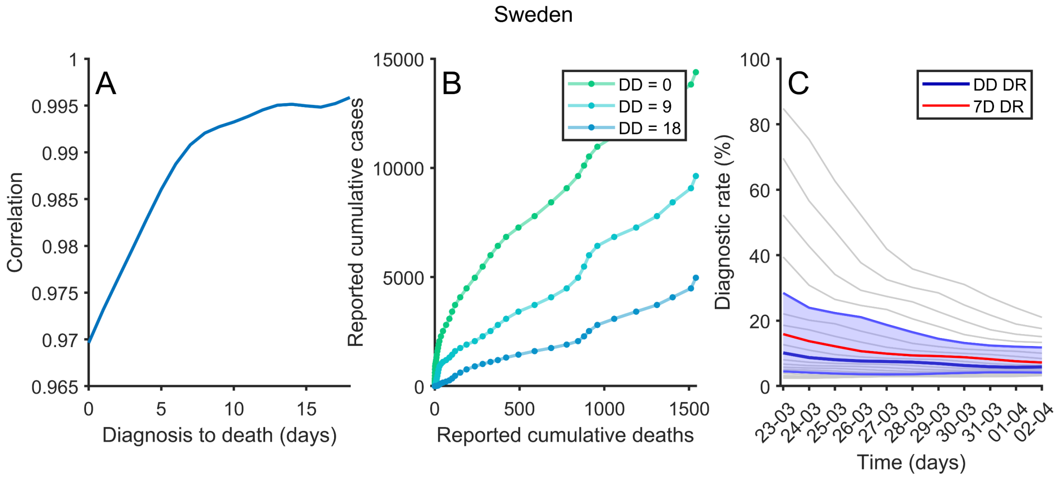

(A) Correlation between reported number of cumulative cases and reported number of cumulative deaths using different DtD times. **(B)** Alignment between reported number of cumulative cases and reported number of cumulative deaths using three different detection delays (DD). **(C)** Diagnostic rate along time using different DD, from top to bottom 0 to 18 days. In red, 7-day detection rate and, in blue, Delay to Detection Diagnostic Rate.

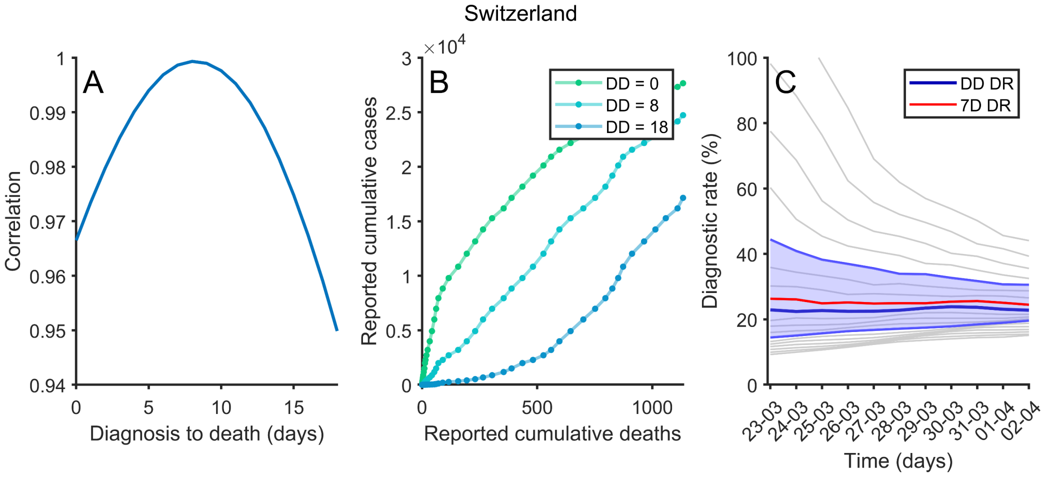

(A) Correlation between reported number of cumulative cases and reported number of cumulative deaths using different DtD times. **(B)** Alignment between reported number of cumulative cases and reported number of cumulative deaths using three different detection delays (DD). **(C)** Diagnostic rate along time using different DD, from top to bottom 0 to 18 days. In red, 7-day detection rate and, in blue, Delay to Detection Diagnostic Rate.

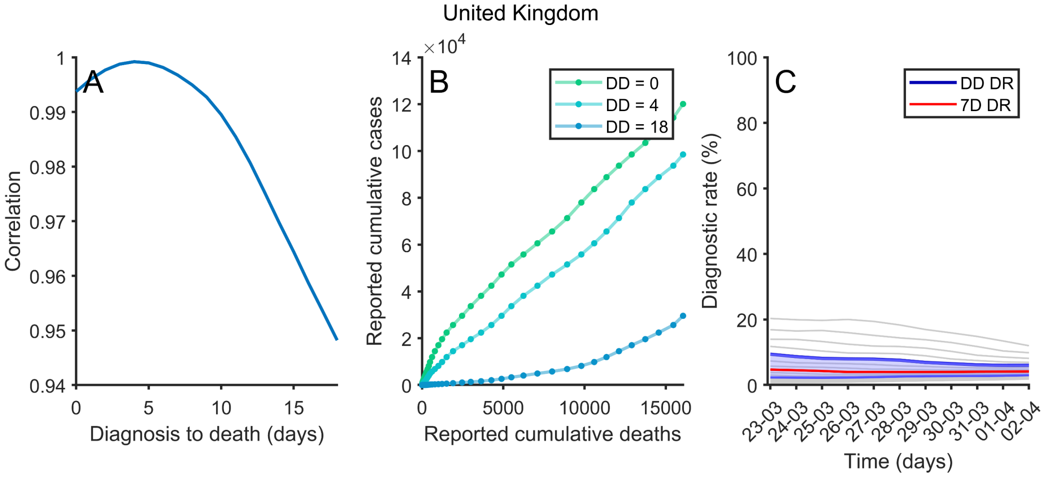

(A) Correlation between reported number of cumulative cases and reported number of cumulative deaths using different DtD times. **(B)** Alignment between reported number of cumulative cases and reported number of cumulative deaths using three different detection delays (DD). **(C)** Diagnostic rate along time using different DD, from top to bottom 0 to 18 days. In red, 7-day detection rate and, in blue, Delay to Detection Diagnostic Rate.

S2 Fig: Evolution of the attack rate and recovered for each country

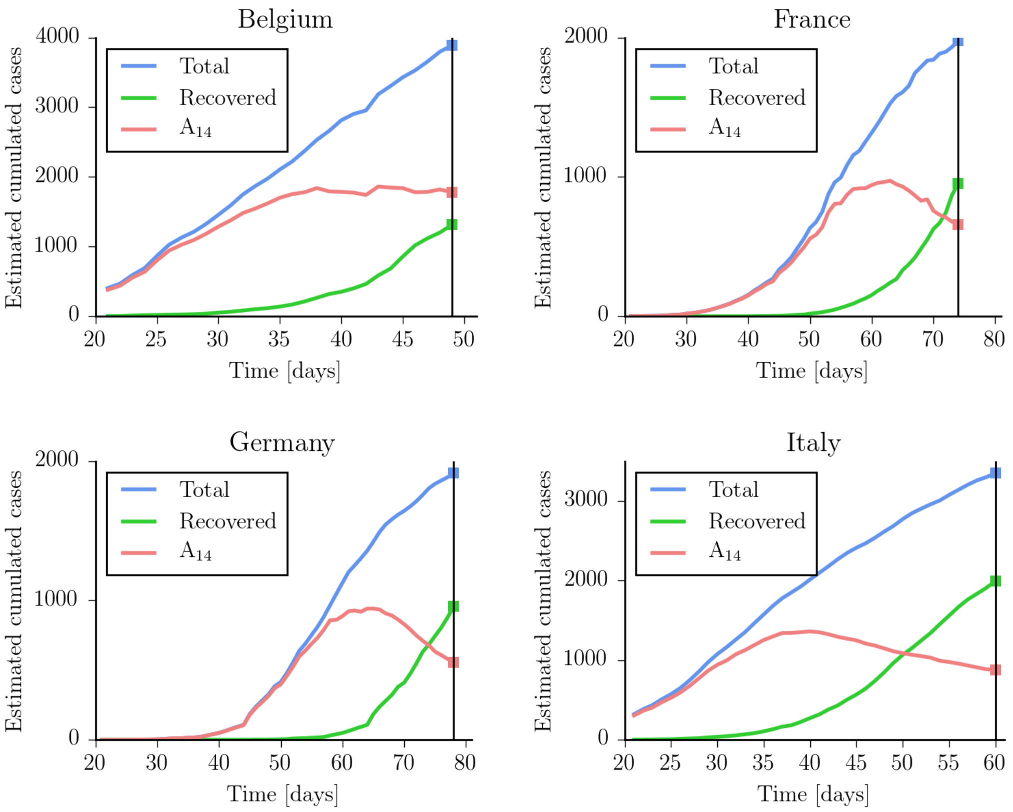

Evolution of the estimated cases for Belgium, France, Germany and Italy . In blue, incidence of estimated cumulative cases. In green, estimated incidence of cumulative recovered cases. In red, estimated incidence of attack rate lasts 14 days (A_{14}). Day 1 is considered the first day where cumulative cases was over 100 cases, Belgium March 4 2020, France February 8 2020, Germany February 4 2020 and Italy February 22 2020 . Data extended till April 20 2020.

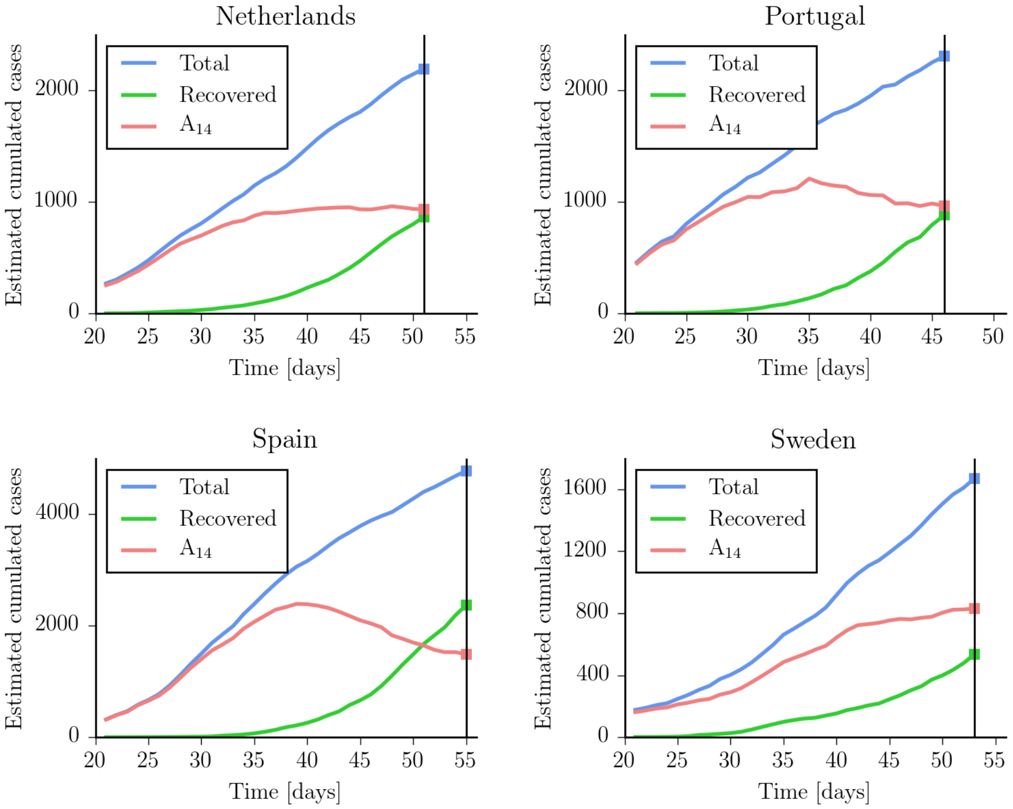

Evolution of the estimated cases for Netherlands, Portugal, Spain and Sweden. In blue, incidence of estimated cumulative cases. In green, estimated incidence of cumulative recovered cases. In red, estimated incidence of attack rate lasts 14 days (A_{14}). Day 1 is considered the first day where cumulative cases was over 100 cases, Netherlands March 2 2020, Portugal March 7 2020, Spain February 27 2020 and Sweden February 29 2020. Data extended till April 20 2020.

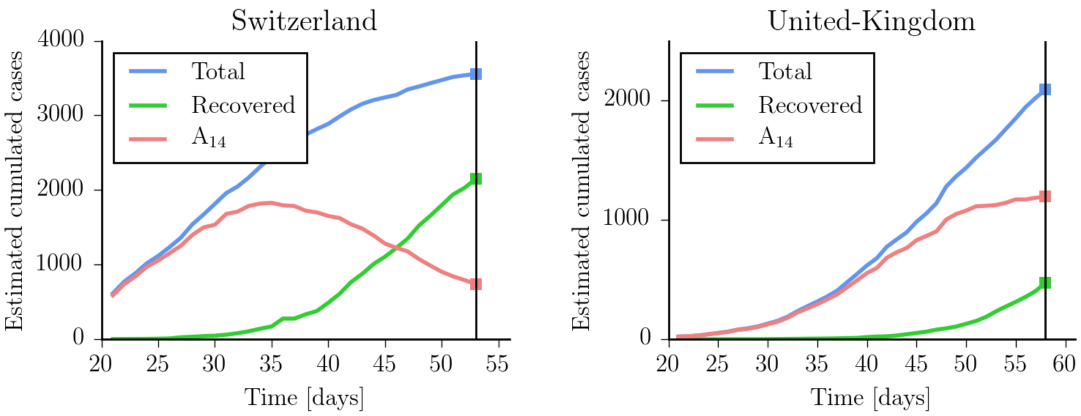

Evolution of the estimated cases for Switzerland and United Kingdom. In blue, incidence of estimated cumulative cases. In green, estimated incidence of cumulative recovered cases. In red, estimated incidence of attack rate lasts 14 days (A_{14}). Day 1 is considered the first day where cumulative cases was over 100 cases, Switzerland February 29 2020 and France February 24 2020 . Data extended till April 20 2020.

S3 Fig: Growth rate of the epidemics. Estimated and reported.

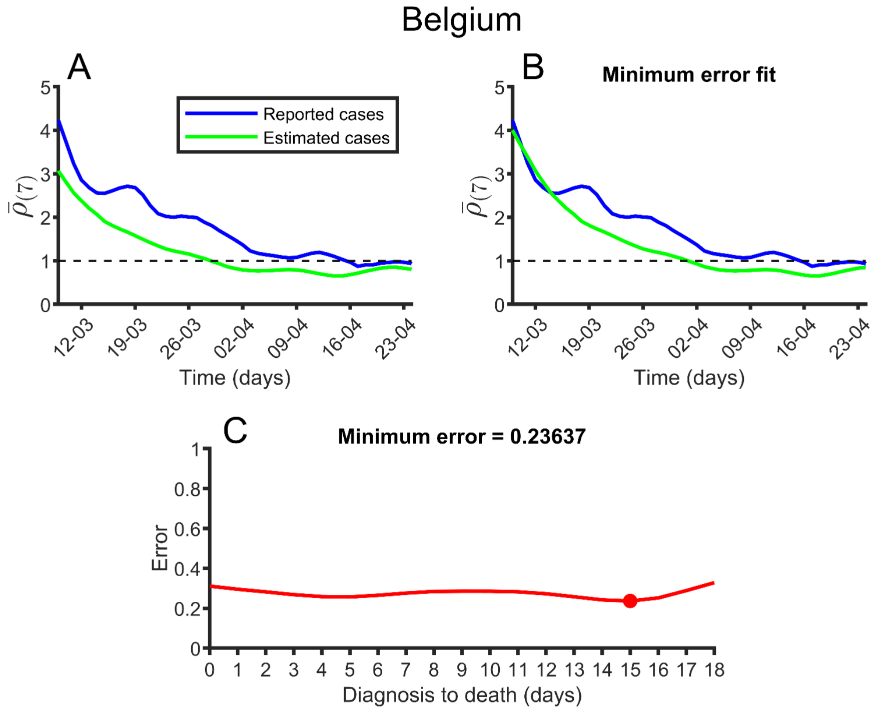

Relation between reported growth rate and estimated growth rate of the epidemic. (A) In green, estimated cases growth rate and, in blue, reported cases growth rate. **(B)** The growth rate of estimated cases is displaced to find better match with the growth rate of reported cases. **(C)** Error between estimated and reported growth rates using different delays. Minimum delay is marked and is the one used in (B).

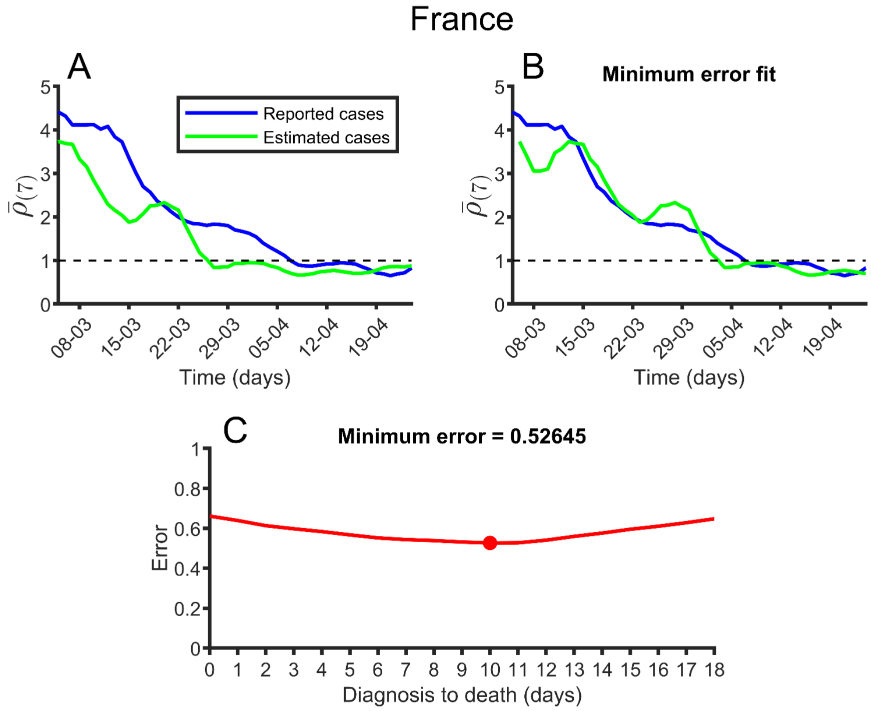

Relation between reported growth rate and estimated growth rate of the epidemic. (A) In green, estimated cases growth rate and, in blue, reported cases growth rate. **(B)** The growth rate of estimated cases is displaced to find better match with the growth rate of reported cases. **(C)** Error between estimated and reported growth rates using different delays. Minimum delay is marked and is the one used in (B)**.**

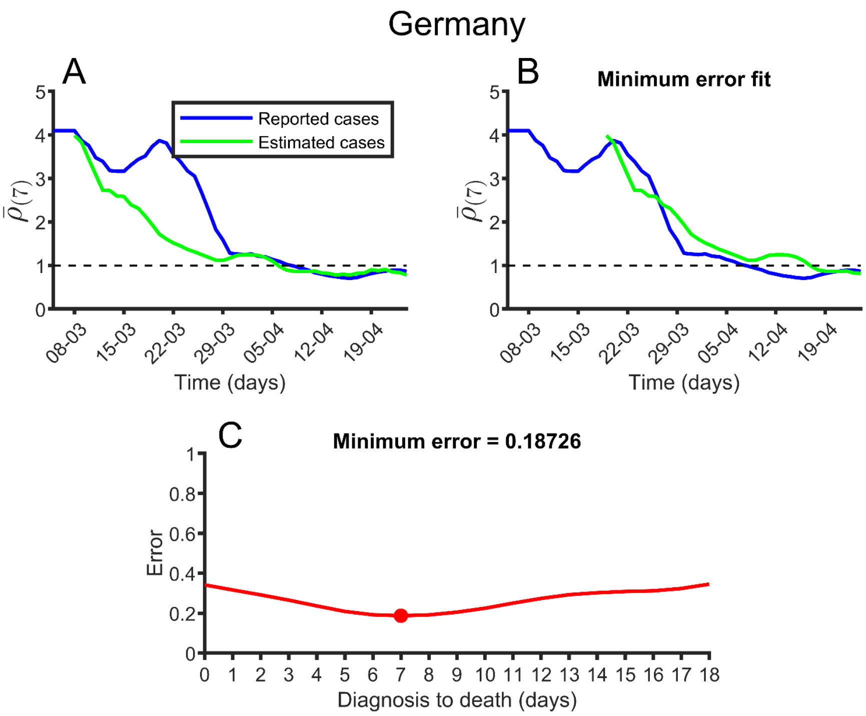

Relation between reported growth rate and estimated growth rate of the epidemic. (A) In green, estimated cases growth rate and, in blue, reported cases growth rate. **(B)** The growth rate of estimated cases is displaced to find better match with the growth rate of reported cases. **(C)** Error between estimated and reported growth rates using different delays. Minimum delay is marked and is the one used in (B)**.**

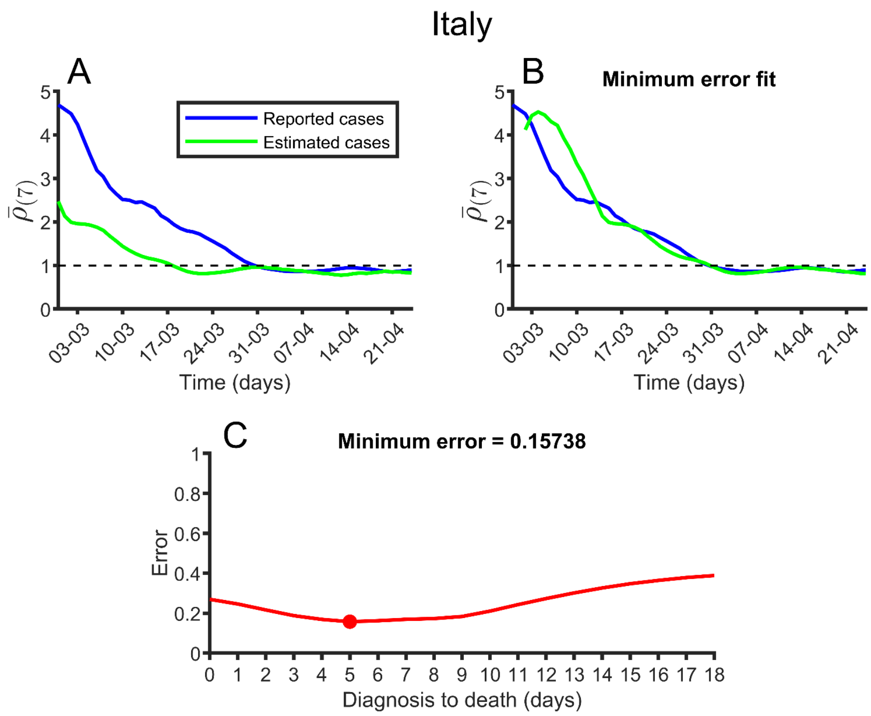

Relation between reported growth rate and estimated growth rate of the epidemic. (A) In green, estimated cases growth rate and, in blue, reported cases growth rate. **(B)** The growth rate of estimated cases is displaced to find better match with the growth rate of reported cases. **(C)** Error between estimated and reported growth rates using different delays. Minimum delay is marked and is the one used in (B).

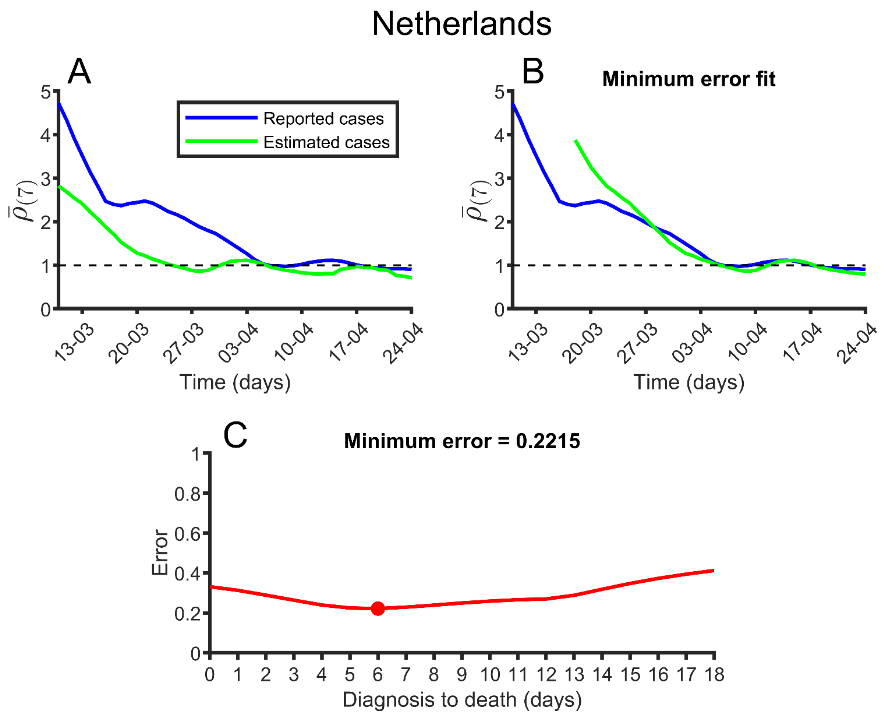

Relation between reported growth rate and estimated growth rate of the epidemic. (A) In green, estimated cases growth rate and, in blue, reported cases growth rate. **(B)** The growth rate of estimated cases is displaced to find better match with the growth rate of reported cases. **(C)** Error between estimated and reported growth rates using different delays. Minimum delay is marked and is the one used in (B)**.**

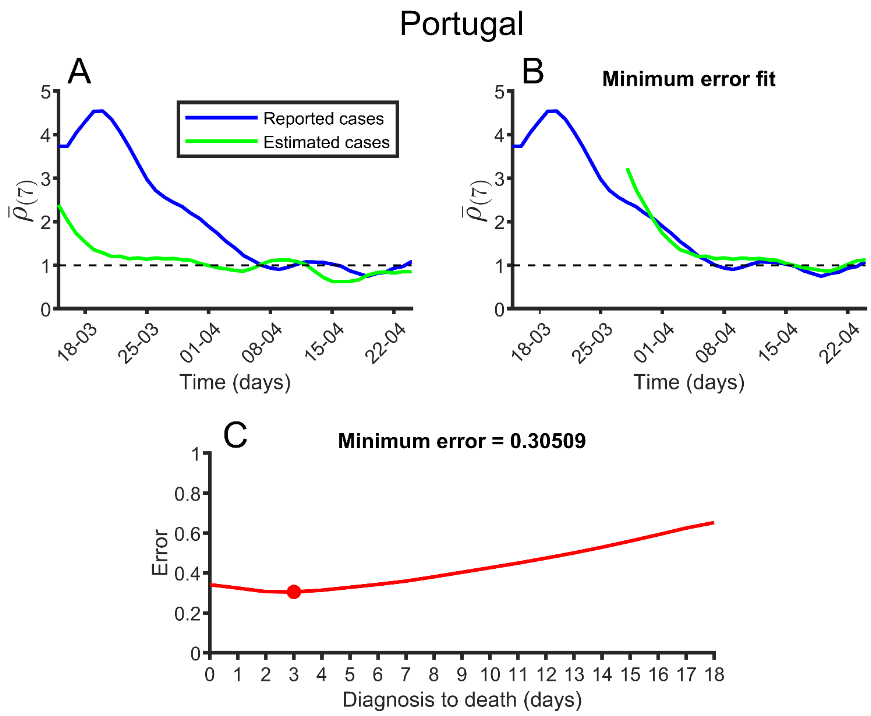

Relation between reported growth rate and estimated growth rate of the epidemic. (A) In green, estimated cases growth rate and, in blue, reported cases growth rate. **(B)** The growth rate of estimated cases is displaced to find better match with the growth rate of reported cases. **(C)** Error between estimated and reported growth rates using different delays. Minimum delay is marked and is the one used in (B)**.**

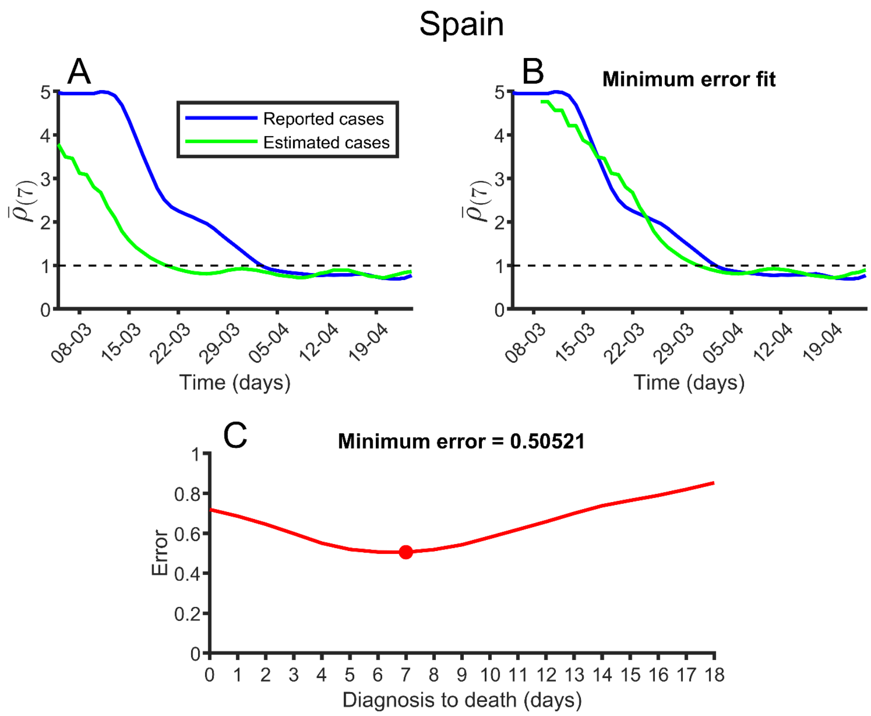

Relation between reported growth rate and estimated growth rate of the epidemic. (A) In green, estimated cases growth rate and, in blue, reported cases growth rate. **(B)** The growth rate of estimated cases is displaced to find better match with the growth rate of reported cases. **(C)** Error between estimated and reported growth rates using different delays. Minimum delay is marked and is the one used in (B)**.**

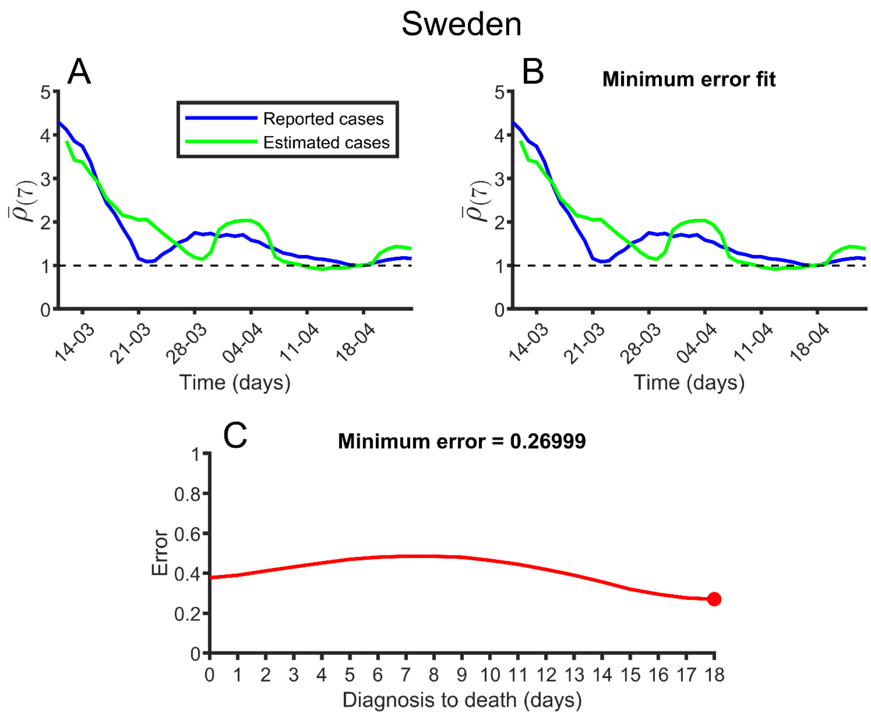

Relation between reported growth rate and estimated growth rate of the epidemic. (A) In green, estimated cases growth rate and, in blue, reported cases growth rate. **(B)** The growth rate of estimated cases is displaced to find better match with the growth rate of reported cases. **(C)** Error between estimated and reported growth rates using different delays. Minimum delay is marked and is the one used in (B)**.**

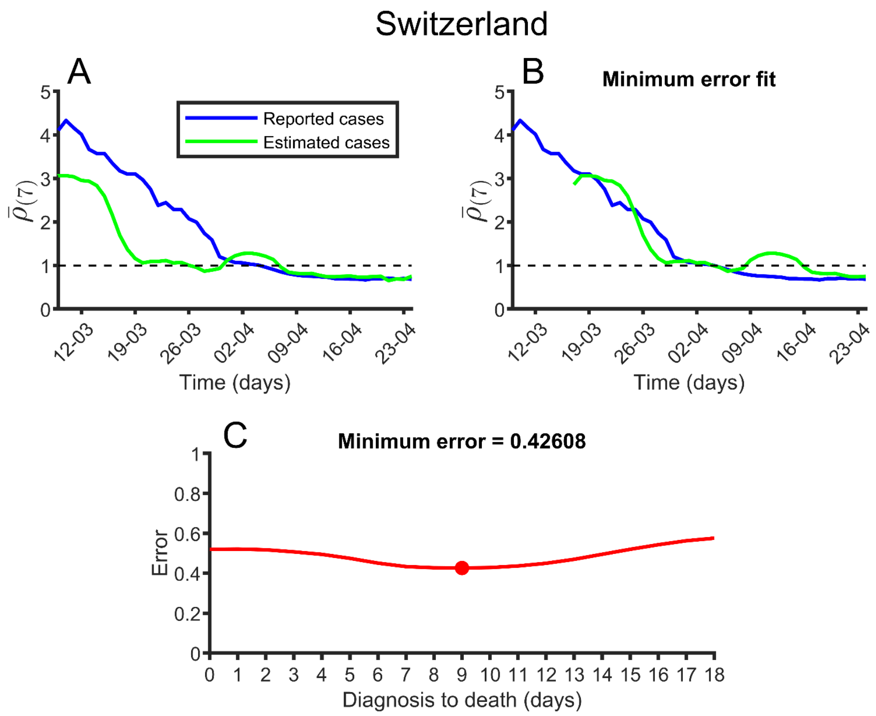

Relation between reported growth rate and estimated growth rate of the epidemic. (A) In green, estimated cases growth rate and, in blue, reported cases growth rate. **(B)** The growth rate of estimated cases is displaced to find better match with the growth rate of reported cases. **(C)** Error between estimated and reported growth rates using different delays. Minimum delay is marked and is the one used in (B).

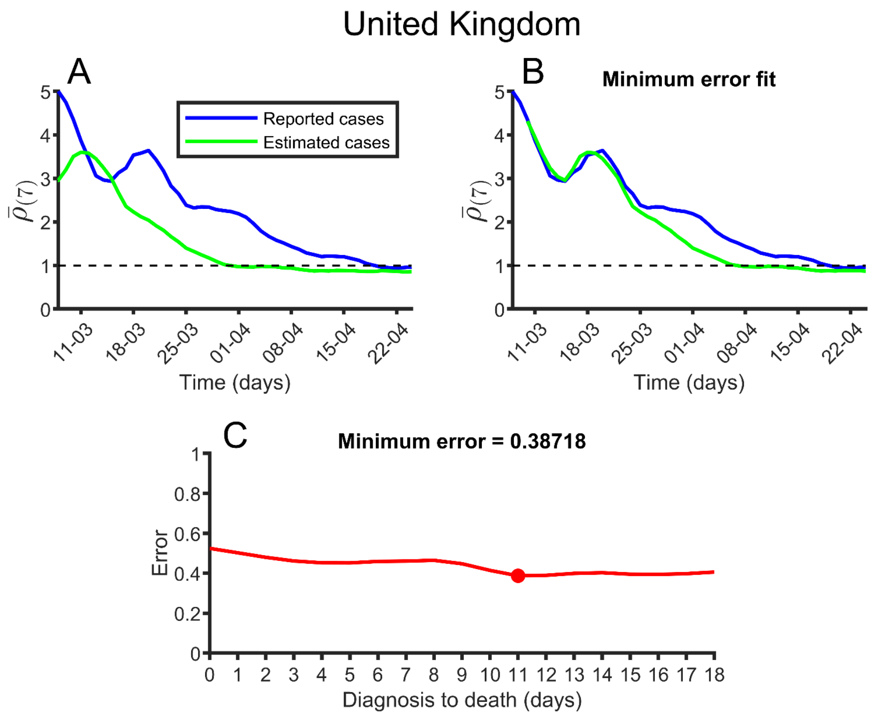

Relation between reported growth rate and estimated growth rate of the epidemic. (A) In green, estimated cases growth rate and, in blue, reported cases growth rate. **(B)** The growth rate of estimated cases is displaced to find better match with the growth rate of reported cases. **(C)** Error between estimated and reported growth rates using different delays. Minimum delay is marked and is the one used in (B)**.**
